# Supplementary material for: Modeling the START transition in the budding yeast cell cycle
Source: PLoS Comput Biol. 2024 Aug 2;20(8):e1012048. doi: 10.1371/journal.pcbi.1012048 (PMC11324117; doi:10.1371/journal.pcbi.1012048)
Supplement: S1 Fig — Initial flow of events in G1 starting from (1) monomers. The top panel is the core model for SBF activation and inactivation as appeared in Fig 2A. The red box containing reactions involved in (2) the complex formation and (3) promoter binding is expanded and shown in the lower panel. (PDF) [file pcbi.1012048.s001.pdf]

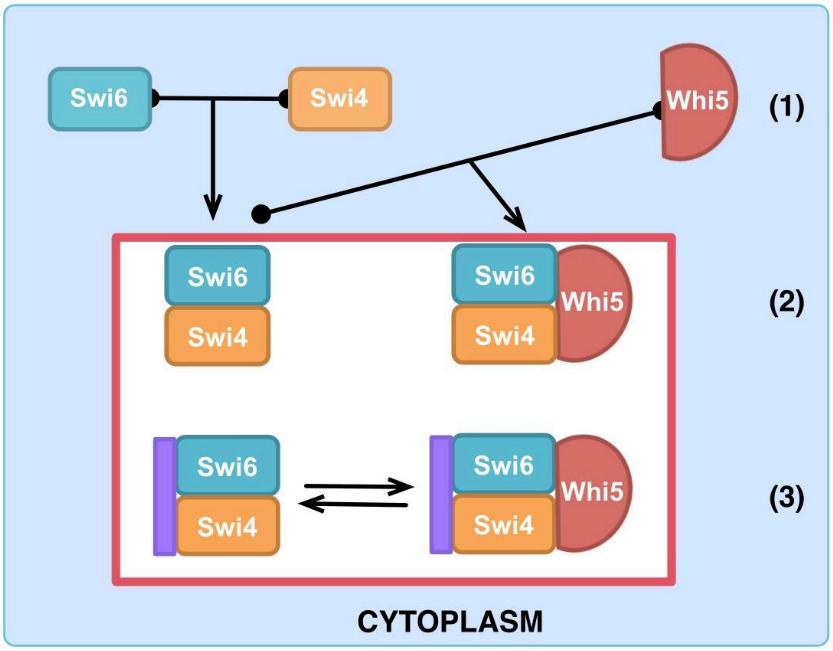

NUCLEUS

Figure S1. Complex formation & promoter binding.

Initial flow of events in G1 starting from (1) monomers. The top panel is the core model for SBF activation and inactivation as appeared in **Figure 2A**. The red box containing reactions involving in (2) the complex formation and (3) promoter binding is expanded and shown in the lower panel.
