# Supplementary material for: Modeling the START transition in the budding yeast cell cycle
Source: PLoS Comput Biol. 2024 Aug 2;20(8):e1012048. doi: 10.1371/journal.pcbi.1012048 (PMC11324117; doi:10.1371/journal.pcbi.1012048)
Supplement: S2 Fig — (i) SBF activation and Whi5 export. In the figure, the cells start with monomers, which proceed to form complexes and bind to the promoter. In WT cells, both Swi6 (P-form) and Whi5 get phosphorylated by Clns (Cln3, Cln1,2 and Clb5,6) and activated by Bck2. We assume that this doubly phosphorylated form is unstable and dissociates to yield active SBF (with Swi6 phosphorylated) and phosphorylated Whi5 that is free to move to the cytoplasm with the help of export protein, Msn5. (ii) SBF inactivation and export. SBF gets inactivated by two sets of Clb phosphorylations (black-filled circles): Q-form on S160 site of Swi6 by Clb5,6 and Clb1,2, and on Swi4 by Clb1,2, leading to dissociation of SBF from promoter. Msn5 recognizes the Q-form of Swi6 phosphorylation (that is in complex with Swi4) for export to the cytoplasm. Complex dissociates in the cytoplasm soon after export. Phosphate groups are indicated as white-filled circles (activatory phosphorylations) or with black-filled circles (inhibitory phosphorylations). (PDF) [file pcbi.1012048.s002.pdf]

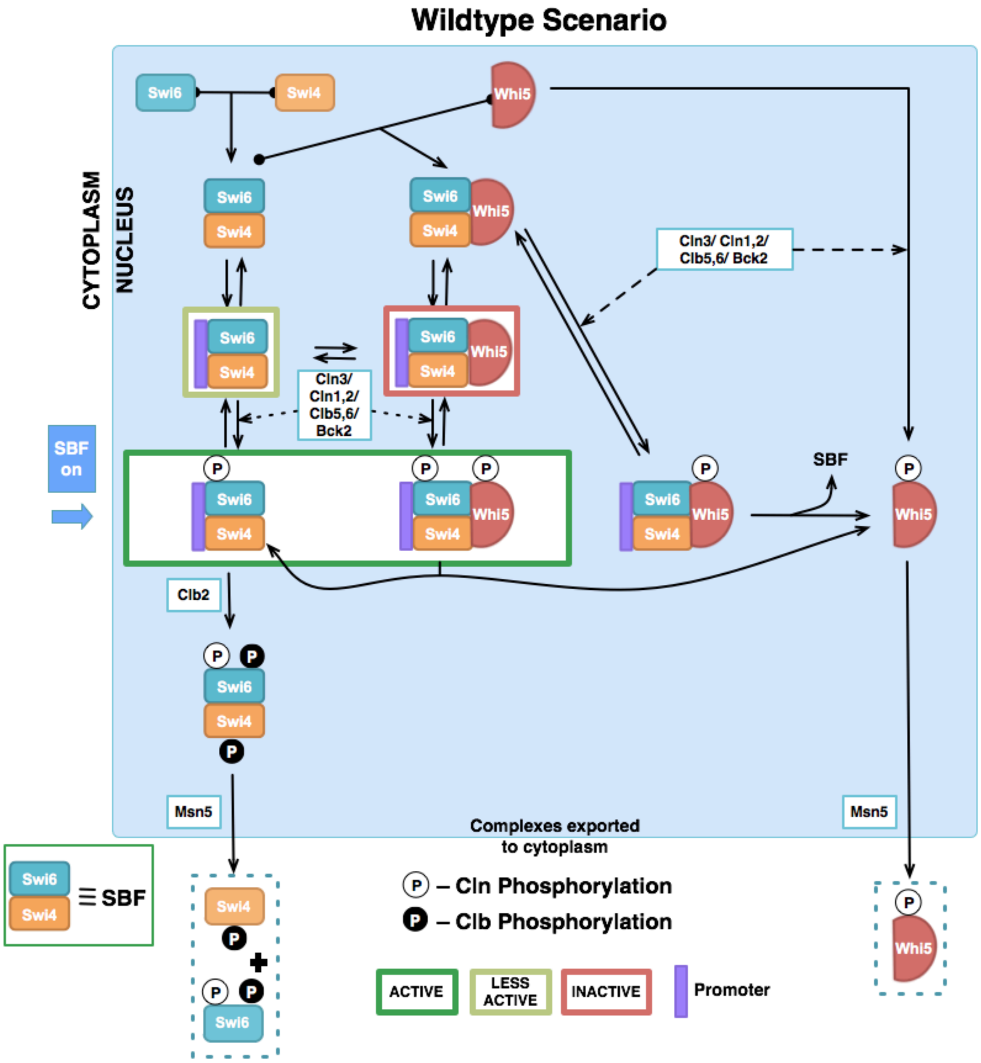

Figure S2. SBF regulation in wildtype cells.

(i) SBF activation and Whi5 export. In the figure, the cells start with monomers, which proceed to form complexes and bind to the promoter. In WT cells, both Swi6 (P-form) and Whi5 get phosphorylated by Clns (Cln3, Cln1,2 and Clb5,6) and activated by Bck2. We assume that this doubly phosphorylated form is unstable and dissociates to yield active SBF (with Swi6 phosphorylated) and phosphorylated Whi5 that is free to move to the cytoplasm with the help of export protein, Msn5. (ii) SBF inactivation and export. SBF gets inactivated by two sets of Clb phosphorylations (black-filled circles): Q-form on S160 site of Swi6 by Clb5,6 and Clb1,2, and on Swi4 by Clb1,2, leading to dissociation of SBF from promoter. Msn5 recognizes the Q-form of Swi6 phosphorylation (that is in complex with Swi4) for export to the cytoplasm. Complex dissociates in the cytoplasm soon after export. Phosphate groups are indicated as white-filled circles (activatory phosphorylations) or with black-filled circles (inhibitory phosphorylations).
