# Supplementary material for: Modeling the START transition in the budding yeast cell cycle
Source: PLoS Comput Biol. 2024 Aug 2;20(8):e1012048. doi: 10.1371/journal.pcbi.1012048 (PMC11324117; doi:10.1371/journal.pcbi.1012048)
Supplement: S3 Fig — We assume that either Clb phosphorylation on Swi6 (S160) (Q-form), or Cln phosphorylation on Whi5 is necessary for recognition by transport protein, Msn5, and subsequent export to the cytoplasm. Additionally, we assume that in promoter-bound complexes, Clb phosphorylation of Swi4 is necessary and sufficient for the complexes to dissociate from the promoter necessary to turn off gene transcription and to facilitate SBF export. Similar to SBF inactivation in S2, SBF Fig complexes dissociate in the cytoplasm soon after export. (PDF) [file pcbi.1012048.s003.pdf]

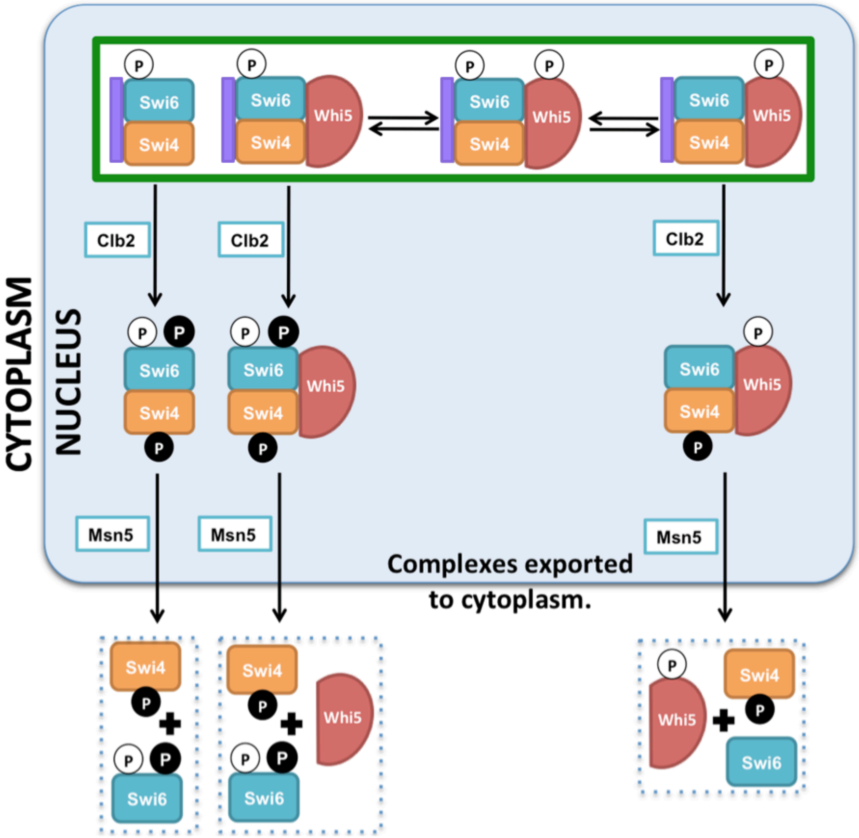

Figure S3. SBF inactivation by Clbs.

We assume that either Clb phosphorylation on Swi6 (S160) (Q-form), or Cln phosphorylation on Whi5 is necessary for recognition by transport protein, Msn5, and subsequent export to the cytoplasm. Additionally, we assume that in promoter-bound complexes, Clb phosphorylation of Swi4 is necessary and sufficient for the complexes to dissociate from the promoter necessary to turn off gene transcription and to facilitate SBF export. Similar to SBF inactivation in Figure S2, SBF complexes dissociate in the cytoplasm soon after export.
