# Supplementary material for: Modeling the START transition in the budding yeast cell cycle
Source: PLoS Comput Biol. 2024 Aug 2;20(8):e1012048. doi: 10.1371/journal.pcbi.1012048 (PMC11324117; doi:10.1371/journal.pcbi.1012048)
Supplement: S4 Fig — The nuclear form of Cln3 is the active form. Nuclear import is controlled by Ydj1 in response to cell size, whereas sequestration of Cln3 in the ER (inhibiting Cln3) is done by Ssa1, probably, in response to late mitotic factors. (See main text for details) We assume that Bck2 is regulated in a similar fashion. (PDF) [file pcbi.1012048.s004.pdf]

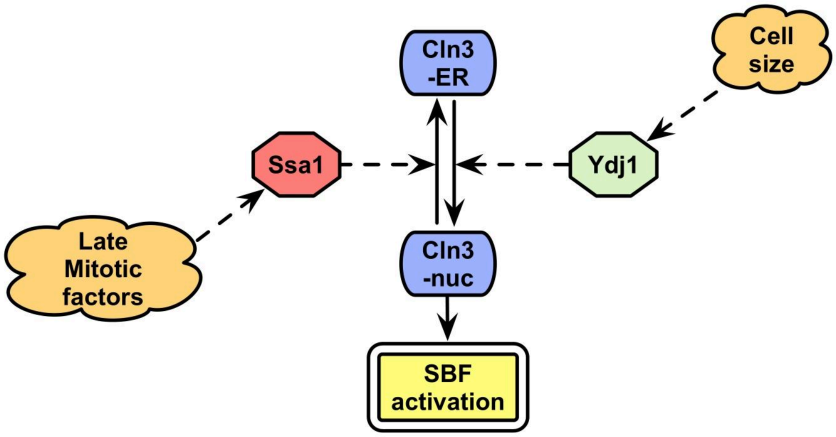

Figure S4. Cartoon of Cln3 regulation by Ydj1/Ssa1.

The nuclear form of Cln3 is the active form. Nuclear import is controlled by Ydj1 in response to cell size, whereas sequestration of Cln3 in the ER (inhibiting Cln3) is done by Ssa1, probably, in response to late mitotic factors. (See main text for details) We assume that Bck2 is regulated in a similar fashion.
