# Supplementary material for: Modeling the START transition in the budding yeast cell cycle
Source: PLoS Comput Biol. 2024 Aug 2;20(8):e1012048. doi: 10.1371/journal.pcbi.1012048 (PMC11324117; doi:10.1371/journal.pcbi.1012048)
Supplement: S5 Fig — The duration plots of daughter cycle times (green-filled squares) and G1 (red-filled triangles) phase versus mass doubling time correspond to the experiments by Lord and Wheals (1980). The budded period (blue-filled circles) was, however, close to 50 and almost constant over the wide range, differing quantitatively from the corresponding curve in the experiments. (PDF) [file pcbi.1012048.s005.pdf]

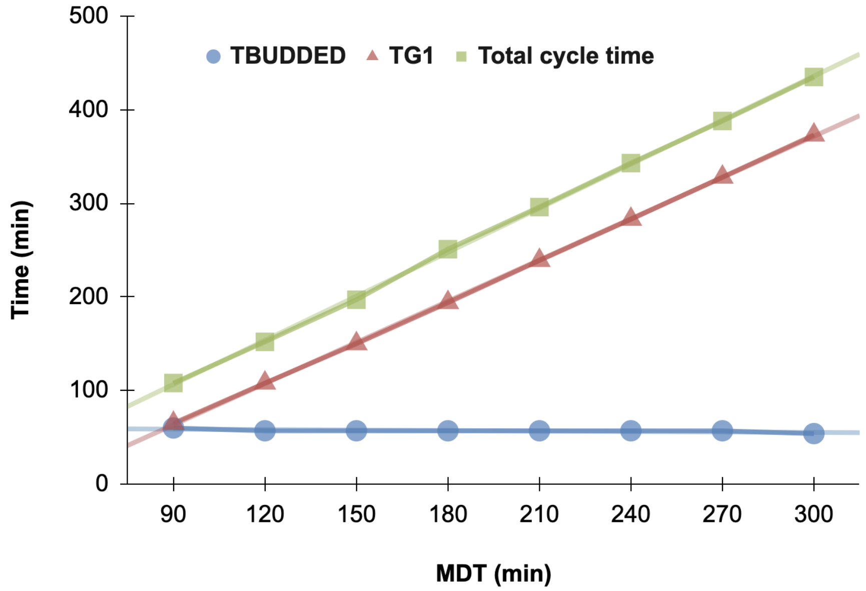

Figure S5. Duration of daughter cycle times as a function of mass doubling time.

The duration plots of daughter cycle times (green-filled squares) and G1 (red-filled triangles) phase versus mass doubling time correspond to the experiments by Lord and Wheals (1980). The budded period (blue-filled circles) was, however, close to 50 and almost constant over the wide range, differing quantitatively from the corresponding curve in the experiments.
