# Supplementary material for: Modeling the START transition in the budding yeast cell cycle
Source: PLoS Comput Biol. 2024 Aug 2;20(8):e1012048. doi: 10.1371/journal.pcbi.1012048 (PMC11324117; doi:10.1371/journal.pcbi.1012048)
Supplement: S6 Fig — This figure describes the timing of re-import and hence overall temporal localization of the different monomers (Swi4, Swi6, Whi5). Export and reimport of Whi5P, although not shown explicitly, follows the same steps as phosphorylated Whi5 in the cartoon. Step (1): SBF complexes that have been phosphorylated on Whi5 or the S160 site of Swi6 are transported to the cytoplasm by Msn5 and dissociate immediately. Phosphorylated Whi5 monomers are also exported by Msn5. Step (2): Unphosphorylated monomers move back to the nucleus (regardless of the phase of the cell cycle). Step (3): Swi4 and the P-form of Swi6 (all phosphorylation sites except S160) get dephosphorylated by PP2A and move to the nucleus. Step (4): the phosphatase Cdc14 that accumulates at mitotic exit dephosphorylates Whi5 and Swi6 Q-form at residue S160, following which Whi5 and Swi6 get reimported to the nucleus resetting the localization state for the G1-phase of the next cell cycle. (PDF) [file pcbi.1012048.s006.pdf]

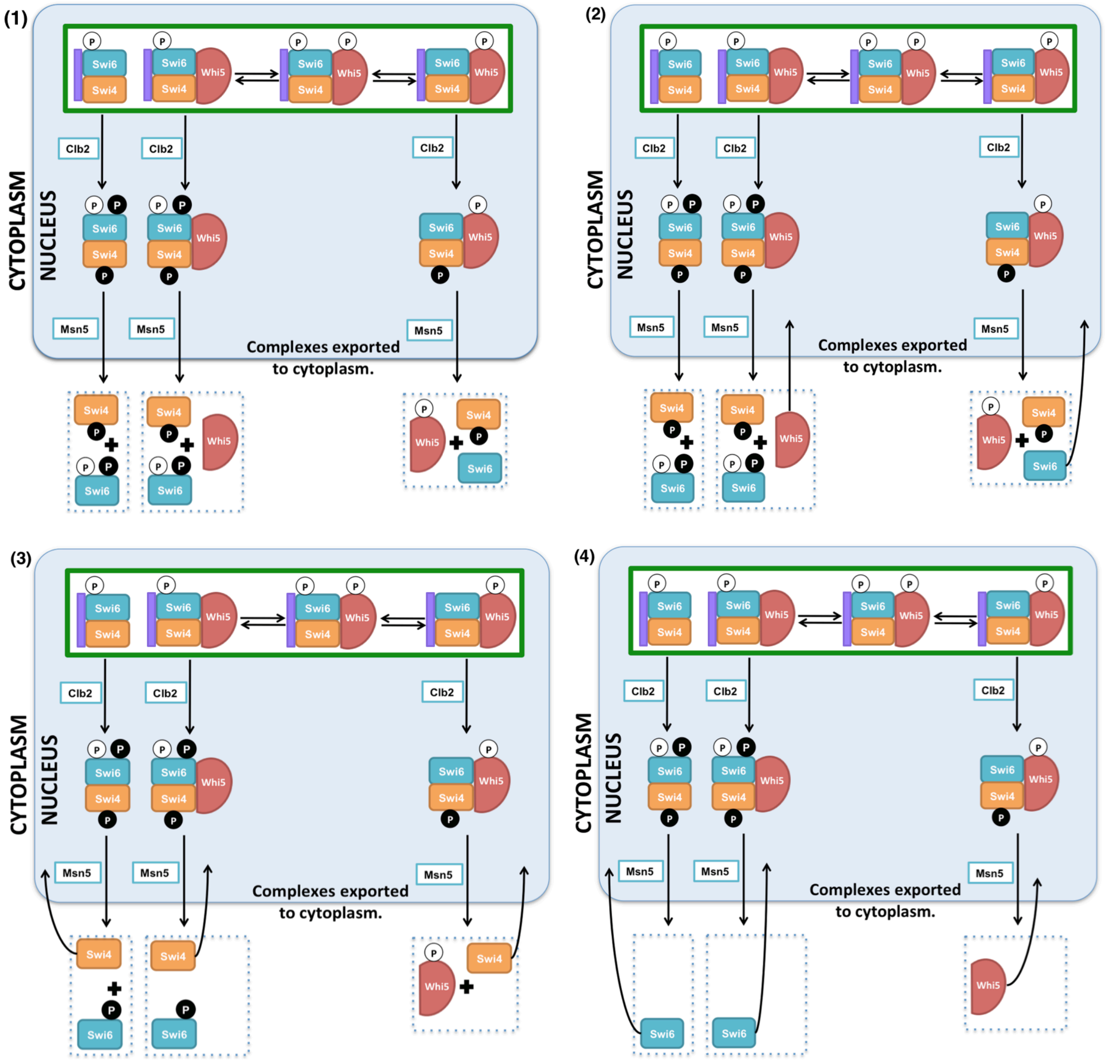

Figure S6. Localization of different monomers.

This figure describes the timing of re-import and hence overall temporal localization of the different monomers (Swi4, Swi6, Whi5). Export and reimport of Whi5P, although not shown explicitly, follows the same steps as phosphorylated Whi5 in the cartoon. Step (1): SBF complexes that have been phosphorylated on Whi5 or the S160 site of Swi6 are transported to the cytoplasm by Msn5 and dissociate immediately. Phosphorylated Whi5 monomers are also exported by Msn5. Step (2): Unphosphorylated monomers move back to the nucleus (regardless of the phase of the cell cycle). Step (3): Swi4 and the P-form of Swi6 (all phosphorylation sites except S160) get dephosphorylated by PP2A and move to the nucleus. Step (4): the phosphatase Cdc14 that accumulates at mitotic exit dephosphorylates Whi5 and Swi6 Q-form at residue S160, following which Whi5 and Swi6 get reimported to the nucleus resetting the localization state for the G1-phase of the next cell cycle.
