# Supplementary material for: Modeling the START transition in the budding yeast cell cycle
Source: PLoS Comput Biol. 2024 Aug 2;20(8):e1012048. doi: 10.1371/journal.pcbi.1012048 (PMC11324117; doi:10.1371/journal.pcbi.1012048)
Supplement: S7 Fig — These simulations are plotted for msn5Δ cells (MSN5 = 0 in our model). The cells are slightly larger than WT cells due to the absence of active SBF, and lesser amount of active MBF (MBFa in the plot above). (PDF) [file pcbi.1012048.s007.pdf]

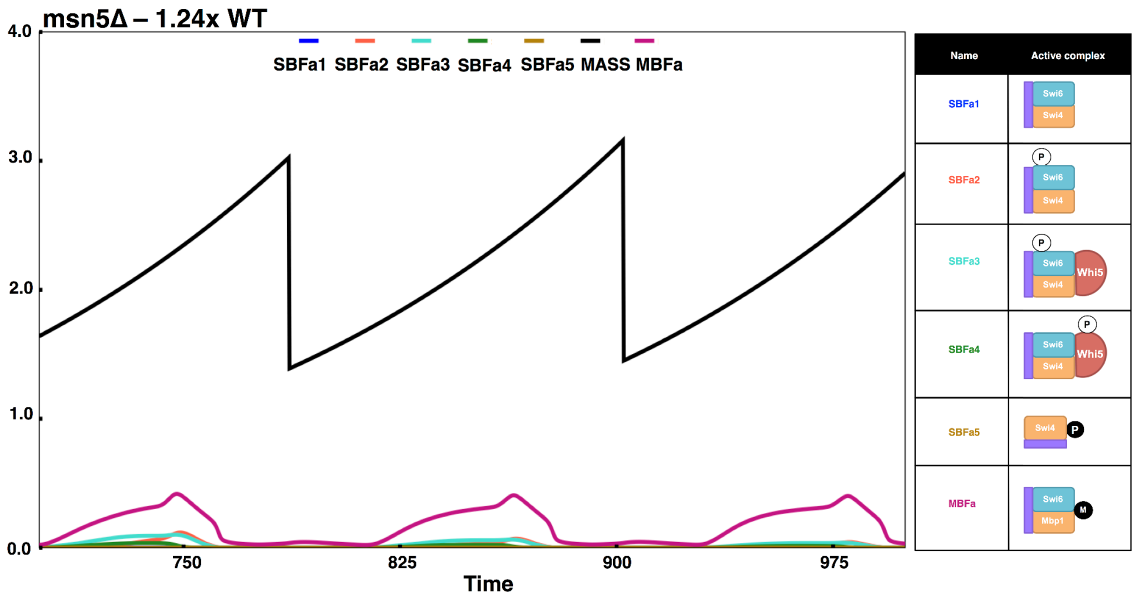

Figure S7. Importance of the transport protein, Msn5.

These simulations are plotted for msn5Δ cells (MSN5=0 in our model). The cells are slightly larger than WT cells due to the absence of active SBF, and lesser amount of active MBF (MBFa in the plot above).
