# Supplementary material for: Modeling the START transition in the budding yeast cell cycle
Source: PLoS Comput Biol. 2024 Aug 2;20(8):e1012048. doi: 10.1371/journal.pcbi.1012048 (PMC11324117; doi:10.1371/journal.pcbi.1012048)
Supplement: S8 Fig — Active complexes in (A) WHI5-12A (This mutant will have size similar to that of WT due to the Swi6 P-forms; Fig 7A), (B) SWI6-SA4 (WT size due to phosphorylation of Whi5; Fig 7B), (C) WHI5-12A SWI6-SA4 (Viable, yet large, due to inactive SBF-Whi5 complex and support from Bck2 activation and MBF; Fig 7C). (PDF) [file pcbi.1012048.s008.pdf]

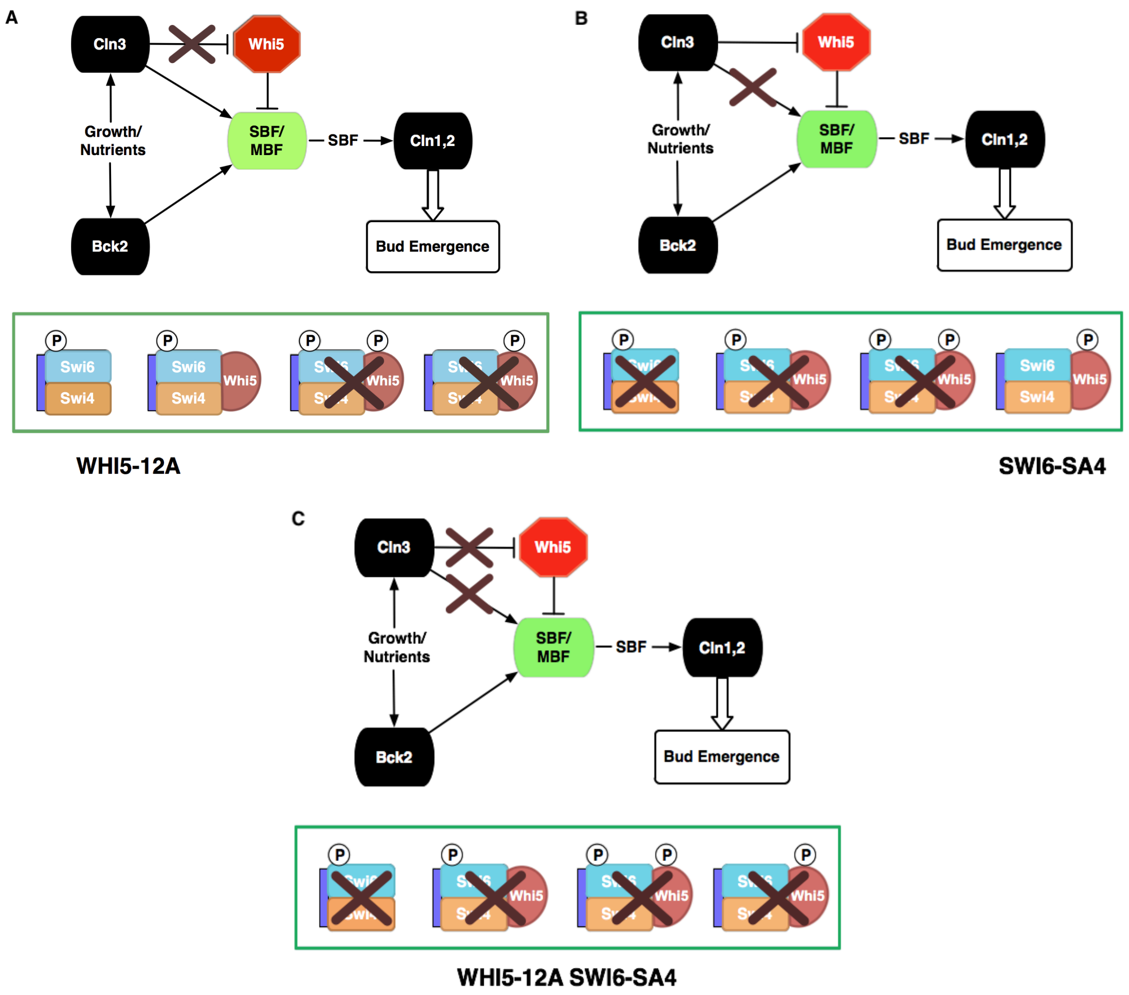

Figure S8. Non-phosphorylable mutants.

Active complexes in (A) *WHI5-12A* (This mutant will have size similar to that of WT due to the Swi6 P-forms; Figure 7A), (B) *SWI6-SA4* (WT size due to phosphorylation of Whi5; Figure 7B), (C) *WHI5-12A SWI6-SA4* (Viable, yet large, due to inactive SBF-Whi5 complex and support from Bck2 activation and MBF; Figure 7C).
