# Supplementary material for: Modeling the START transition in the budding yeast cell cycle
Source: PLoS Comput Biol. 2024 Aug 2;20(8):e1012048. doi: 10.1371/journal.pcbi.1012048 (PMC11324117; doi:10.1371/journal.pcbi.1012048)
Supplement: S9 Fig — The figure shows simulations of the following mutants: (A) swi6Δ GAL-WHI5 (only Swi4dimers present (SBFa5); cells are viable yet large), (B) swi4Δ (only MBF present; cells are viable yet large), (C) msn5Δ swi4Δ (only MBF present; cells are viable and large), (D) swi6Δ (only Swi4dimers (SBFa5) present; cells are viable yet large), (E) msn5Δ swi6Δ (only Swi4dimers (SBFa5) present; cells are viable yet large), (F) cln1Δ cln2Δ cdh1Δ (cells are inviable), (G) cln1Δ cln2Δ cdh1Δ GAL-CLN2 (cells are viable, very small). A, C, E, F and G are in contradiction with experimental findings, while (H) cln1Δ cln2Δ cdh1Δ GAL-CLN2 GAL-SIC (cells are viable) is complementary with our hypothesis about the effect of Cdh1 on Polo and Cdc14 on cell viability due to higher CKI levels. (PDF) [file pcbi.1012048.s009.pdf]

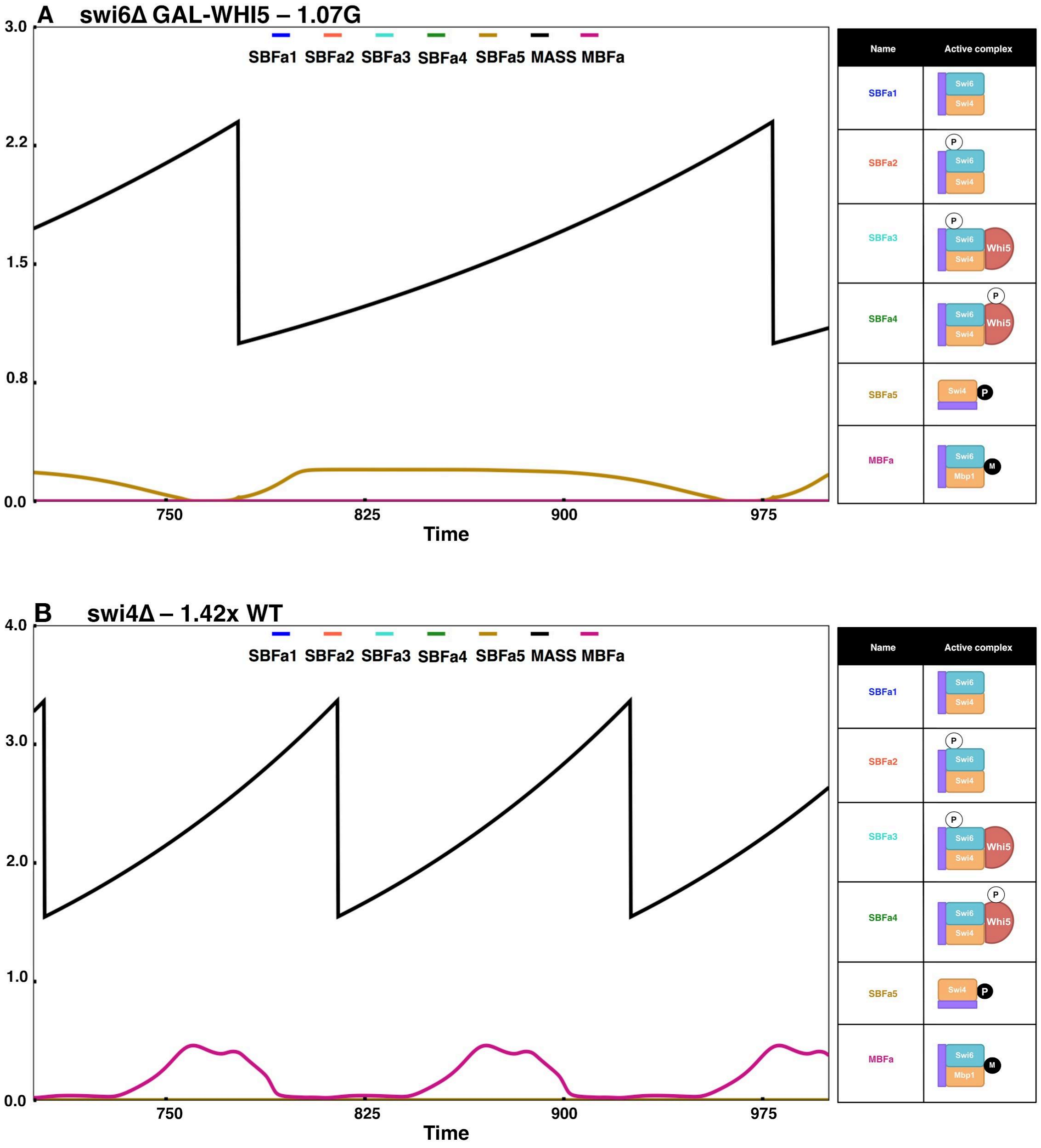
A swi6Δ GAL-WHI5 – 1.07G
SBFa1 SBFa2 SBFa3 SBFa4 SBFa5 MASS MBFa
3.0
2.2
1.5
0.8
0.0
750
825
900
975
Time
Name
Active complex
SBFa1
SBFa2
SBFa3
SBFa4
SBFa5
MBFa
Swi6
Swi4
P
Whi5
Mbp1
M
B swi4Δ – 1.42x WT
SBFa1 SBFa2 SBFa3 SBFa4 SBFa5 MASS MBFa
4.0
3.0
2.0
1.0
0.0
750
825
900
975
Time
Name
Active complex
SBFa1
SBFa2
SBFa3
SBFa4
SBFa5
MBFa

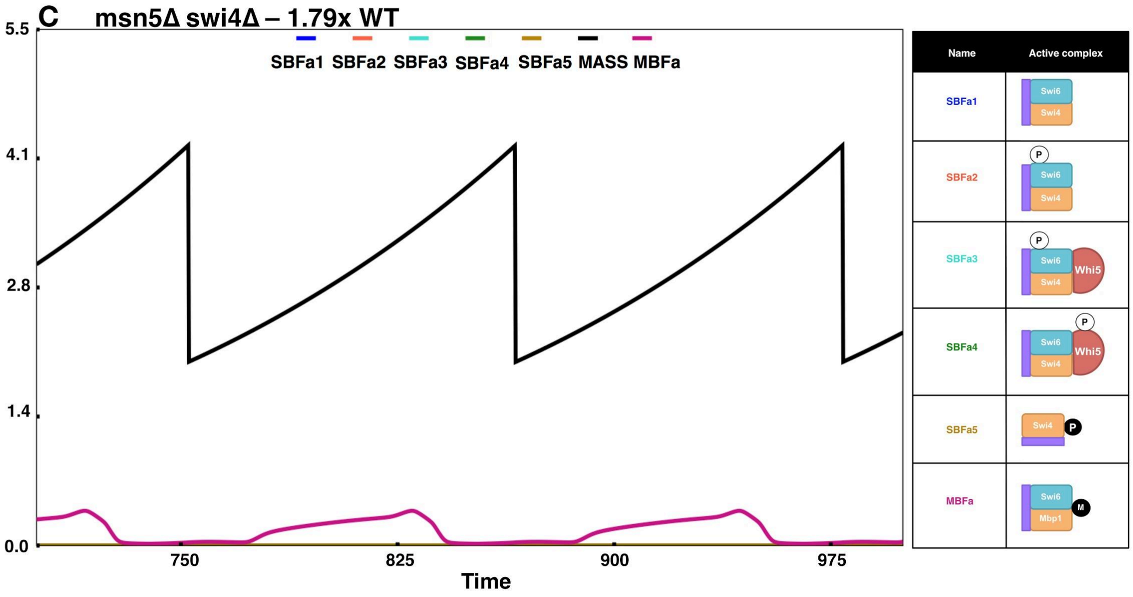
C
msn5Δ swi4Δ – 1.79x WT
SBFa1 SBFa2 SBFa3 SBFa4 SBFa5 MASS MBFa
5.5
4.1
2.8
1.4
0.0
750
825
900
975
Time
Name
Active complex
SBFa1
SBFa2
SBFa3
SBFa4
SBFa5
MBFa
Swi6
Swi4
Whi5
Mbp1
P
M

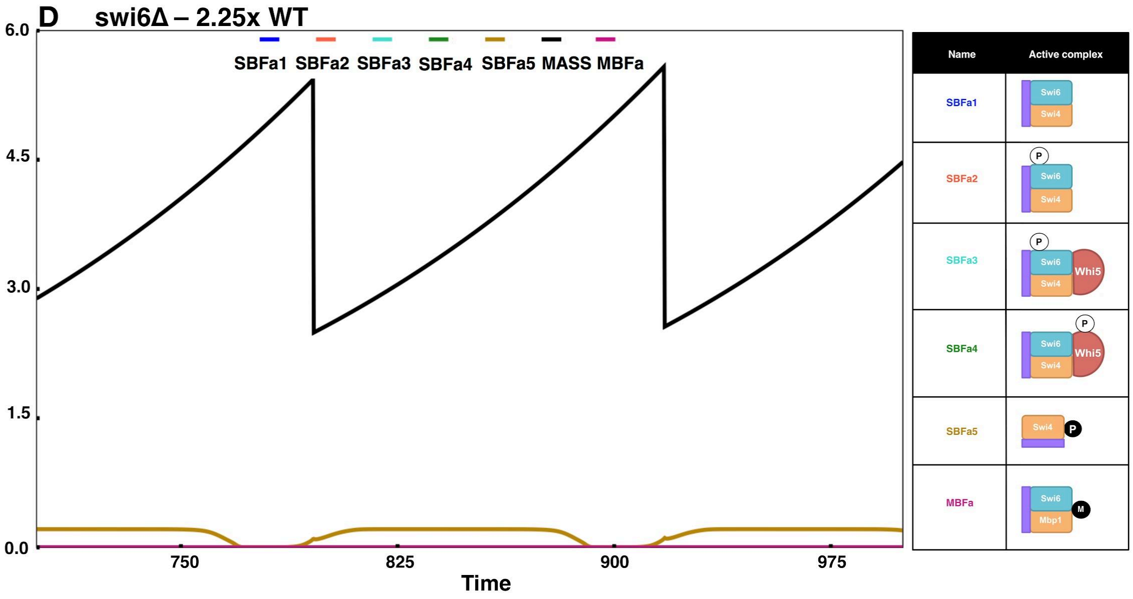
D
swi6Δ – 2.25x WT
SBFa1 SBFa2 SBFa3 SBFa4 SBFa5 MASS MBFa
6.0
4.5
3.0
1.5
0.0
750
825
900
975
Time
Name
Active complex
SBFa1
SBFa2
SBFa3
SBFa4
SBFa5
MBFa
Swi6
Swi4
Whi5
Mbp1
P
M

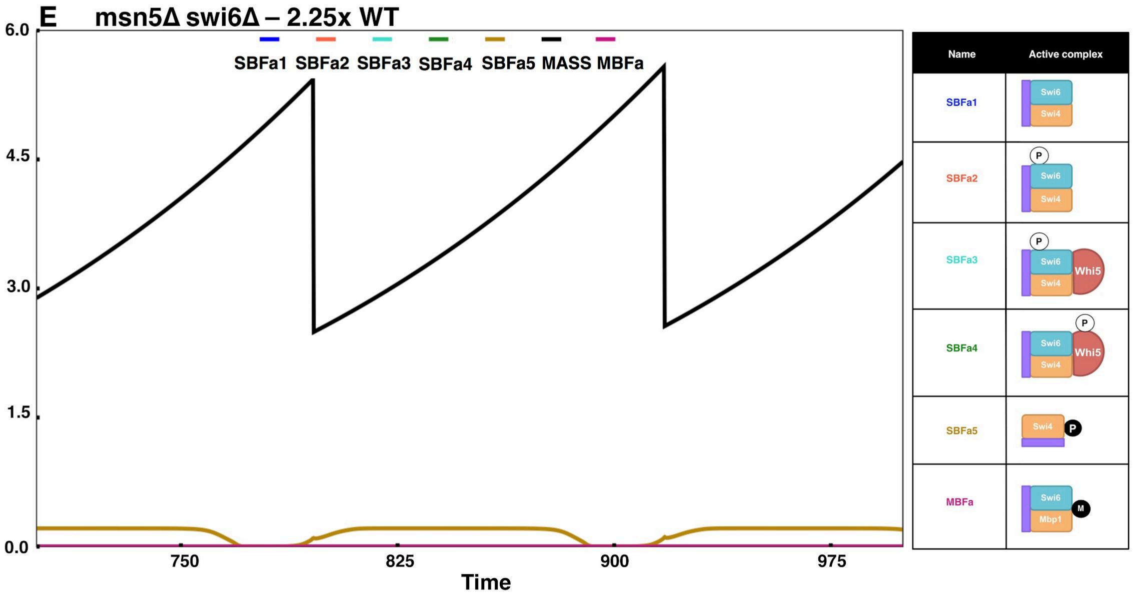
E
msn5Δ swi6Δ – 2.25x WT
SBFa1 SBFa2 SBFa3 SBFa4 SBFa5 MASS MBFa
6.0
4.5
3.0
1.5
0.0
750
825
900
975
Time
Name
Active complex
SBFa1
SBFa2
SBFa3
SBFa4
SBFa5
MBFa
Swi6
Swi4
Whi5
Mbp1
P
M

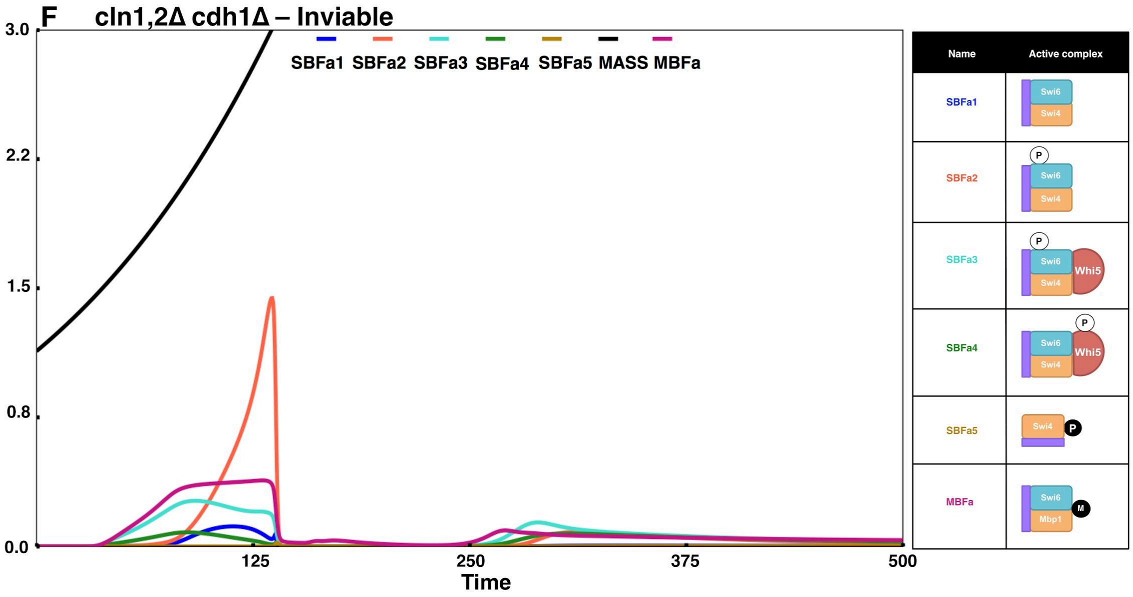
F
cln1,2Δ cdh1Δ – Inviable
SBFa1 SBFa2 SBFa3 SBFa4 SBFa5 MASS MBFa
3.0
2.2
1.5
0.8
0.0
125
250
375
500
Time
Name
Active complex
SBFa1
SBFa2
SBFa3
SBFa4
SBFa5
MBFa
Swi6
Swi4
Whi5
Mbp1
P
M

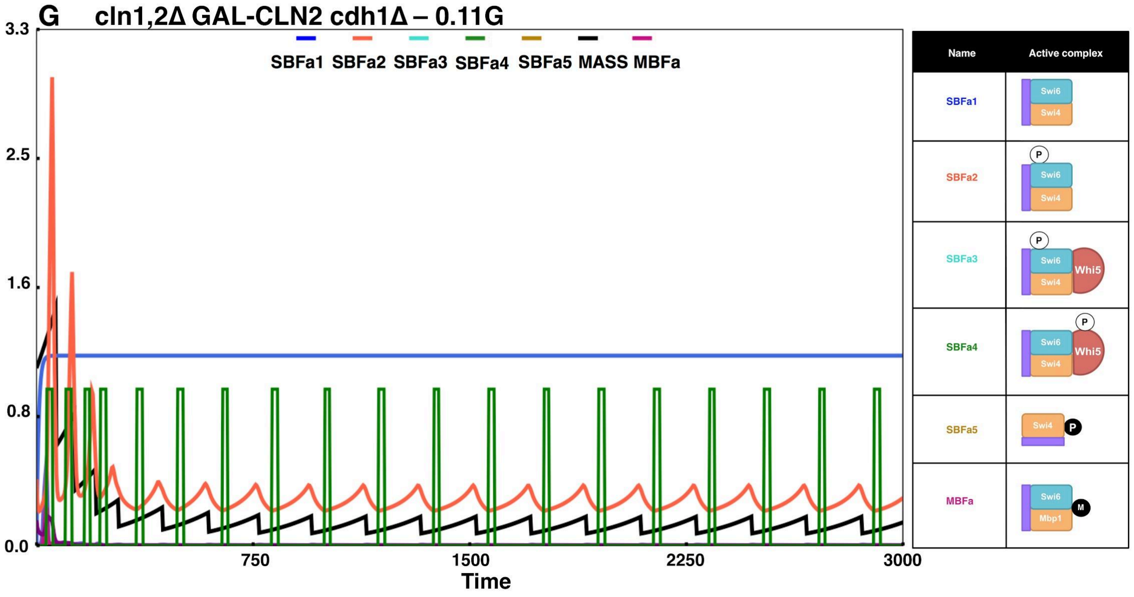

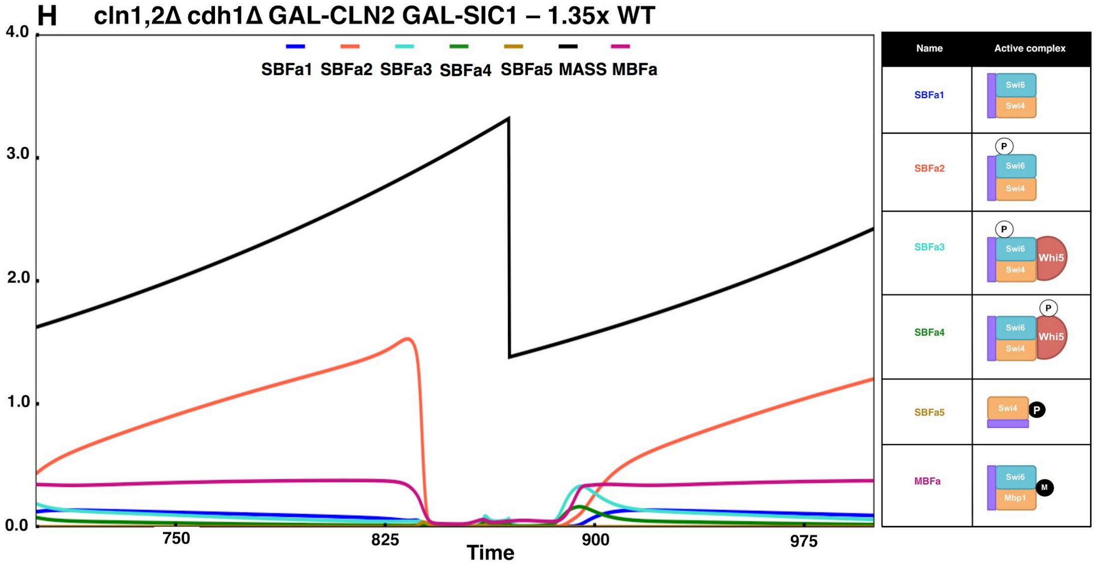

S9 Figure. Model contradictions.

The figure shows simulations of the following mutants: (A) *swi6Δ GAL-WHI5* (only Swi4dimers present (SBFa5); cells are viable yet large), (B) *swi4Δ* (only MBF present; cells are viable yet large), (C) *msn5Δ swi4Δ* (only MBF present; cells are viable and large), (D) *swi6Δ* (only Swi4dimers (SBFa5) present; cells are viable yet large), (E) *msn5Δ swi6Δ* (only Swi4dimers (SBFa5) present; cells are viable yet large), (F) *cln1Δ cln2Δ cdh1Δ* (cells are inviable), (G) *cln1Δ cln2Δ cdh1Δ GAL-CLN2* (cells are viable, very small). A, C, E, F and G are in contradiction with experimental findings, while (H) *cln1Δ cln2Δ cdh1Δ GAL-CLN2 GAL-SIC (cells are viable) is complementary with our hypothesis about the effect of* Cdh1 on Polo and Cdc14 on cell viability due to higher CKI levels*.*
