# Supplementary material for: Modeling the START transition in the budding yeast cell cycle
Source: PLoS Comput Biol. 2024 Aug 2;20(8):e1012048. doi: 10.1371/journal.pcbi.1012048 (PMC11324117; doi:10.1371/journal.pcbi.1012048)
Supplement: S10 Fig — (A) bck2Δ mbp1Δ (Cln-activated forms of SBF are present in varying fractions (SBFa2>SBFa3, SBFa4> SBFa1); cells are viable), (B) bck2Δ mbp1Δ GAL-WHI5 (Cln-activated forms of SBF are present (SBFa2, SBFa3, SBFa4) even if they are lesser than in A; cells are still viable), (C) cln3Δ mbp1Δ (SBFa2 > SBFa3 >> SBFa4 > SBFa5; no MBF is present; cells are viable), (D) cln3Δ mbp1Δ swi6Δ (Swi4dimers (SBFa5) are present sufficiently enough to rescue cells; cells are viable), (E) cln3Δ mbp1Δ whi5Δ (Inhibition on SBF is relieved); cells are rescued), (F) cln3Δ swi4Δ whi5Δ (More Bck2-activated MBF is present (MBFa), there’s no SBF; cells are viable), (G) cln3Δ swi4Δ GAL-BCK2 (There’s a sufficient amount of Bck2-activated MBF (MBFa); cells are rescued), (H) mbp1Δ GAL-WHI5 cells are viable since Whi5 is predicted to get activated and is present in the forms of SBFa2, SBFa3, and SBFa4 (I) mbp1Δ GAL-WHI5-12A cells are viable because Whi5 get converted to the activated form SBFa3. (PDF) [file pcbi.1012048.s010.pdf]

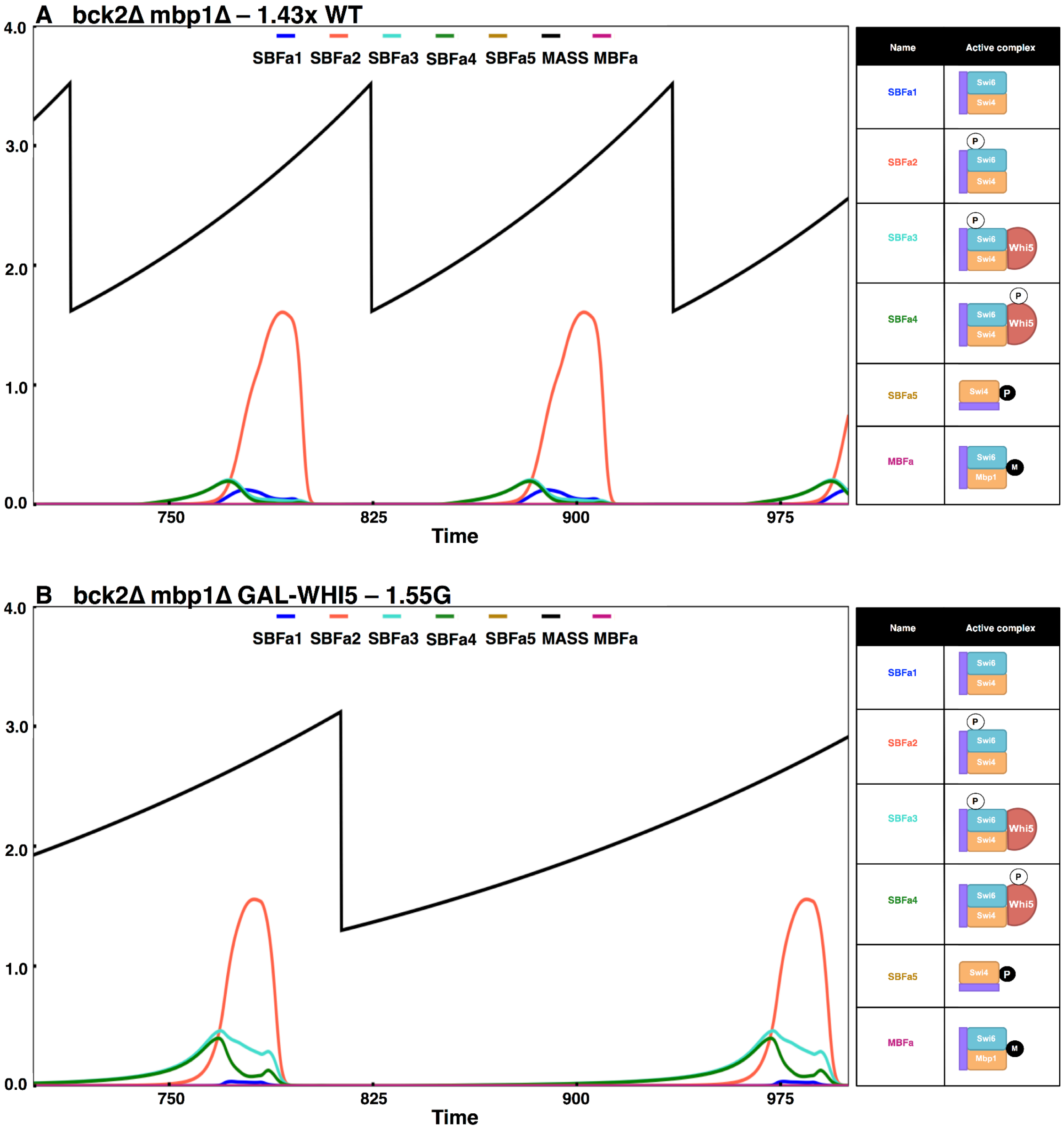
A bck2Δ mbp1Δ – 1.43x WT
B bck2Δ mbp1Δ GAL-WHI5 – 1.55G
SBFa1 SBFa2 SBFa3 SBFa4 SBFa5 MASS MBFa
4.0
3.0
2.0
1.0
0.0
750
825
900
975
Time
Name
Active complex
SBFa1
SBFa2
SBFa3
SBFa4
SBFa5
MBFa
Swi6
Swi4
Whi5
Mbp1
P
M

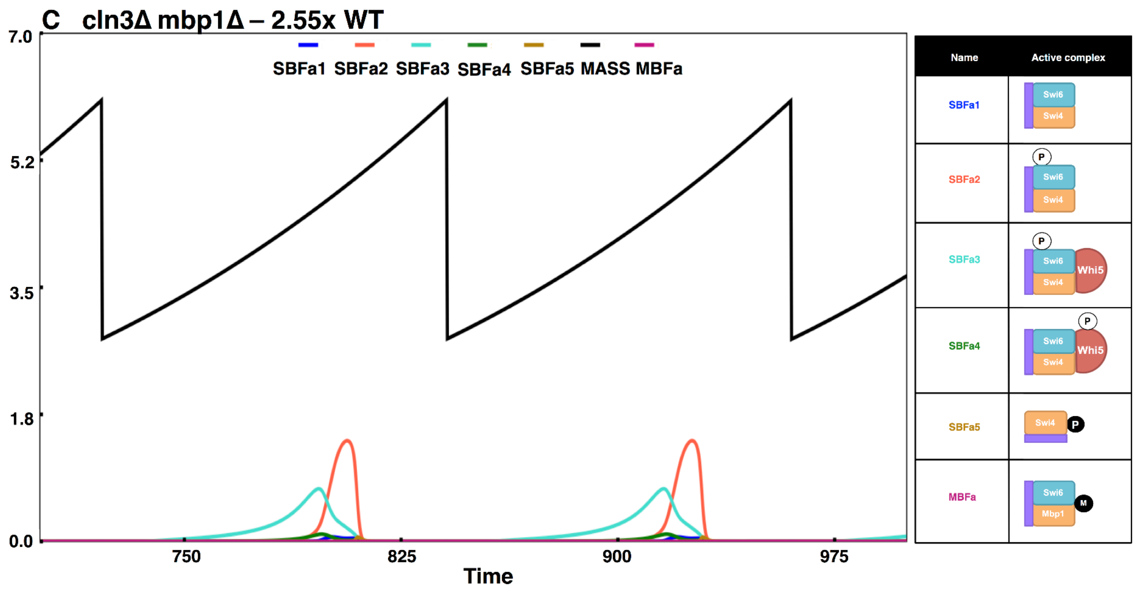
C cln3Δ mbp1Δ – 2.55x WT
7.0
5.2
3.5
1.8
0.0
SBFa1 SBFa2 SBFa3 SBFa4 SBFa5 MASS MBFa
750
825
900
975
Time
Name
Active complex
SBFa1
SBFa2
SBFa3
SBFa4
SBFa5
MBFa
Swi6
Swi4
P
Whi5
M
Mbp1

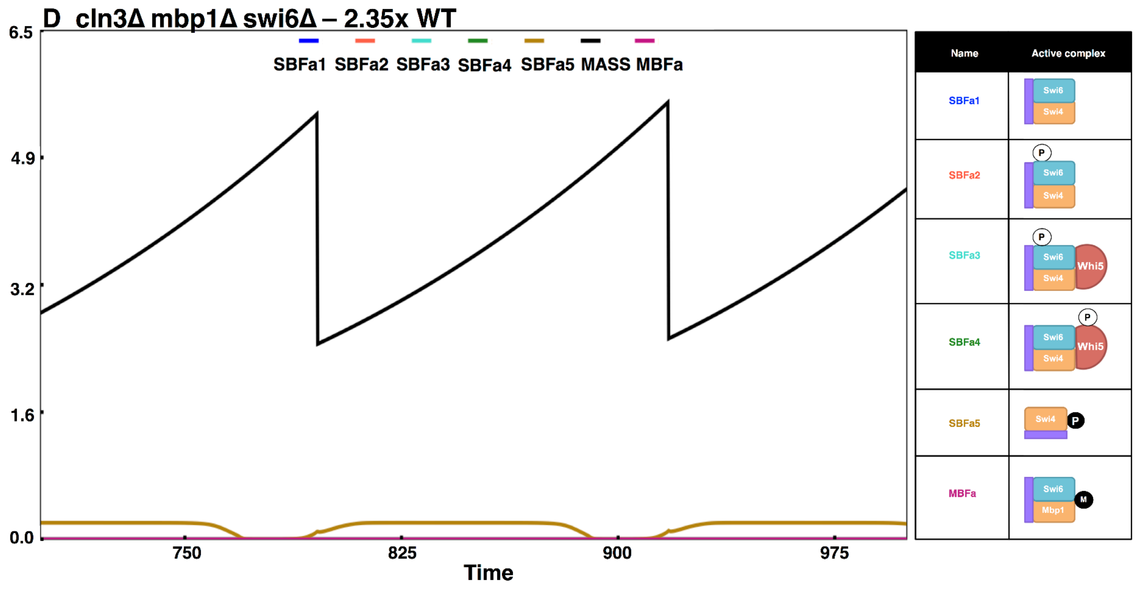
D cln3Δ mbp1Δ swi6Δ – 2.35x WT
6.5
4.9
3.2
1.6
0.0
SBFa1 SBFa2 SBFa3 SBFa4 SBFa5 MASS MBFa
750
825
900
975
Time
Name
Active complex
SBFa1
SBFa2
SBFa3
SBFa4
SBFa5
MBFa
Swi6
Swi4
P
Whi5
M
Mbp1

E cln3Δ mbp1Δ whi5Δ – 1.27x WT

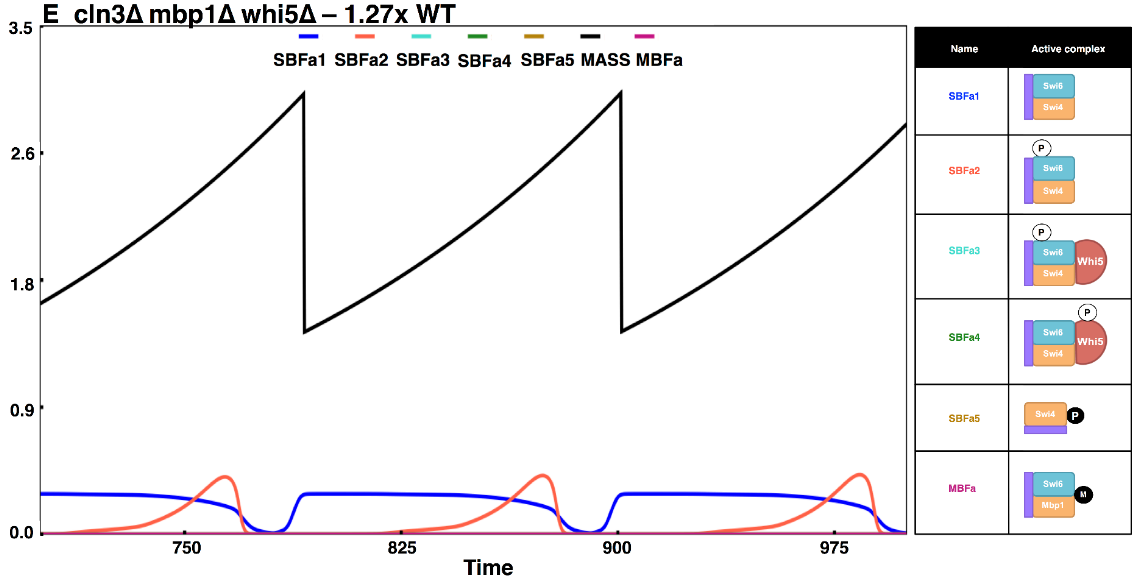


F cln3Δ swi4Δ whi5Δ – 2.48x WT

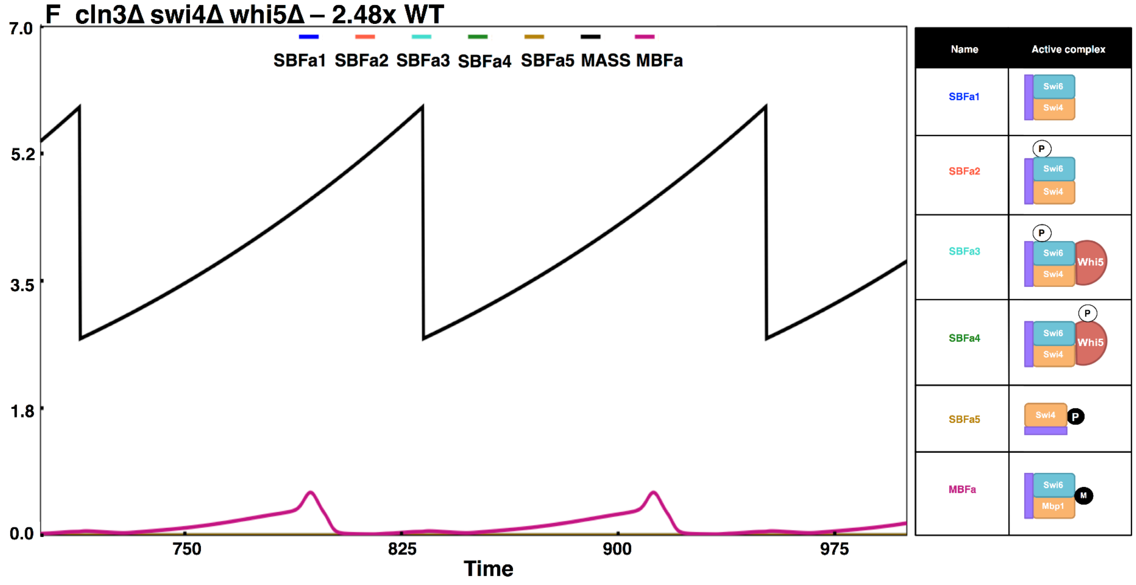

G cln3Δ swi4Δ GAL-BCK2 – 1.09x WT

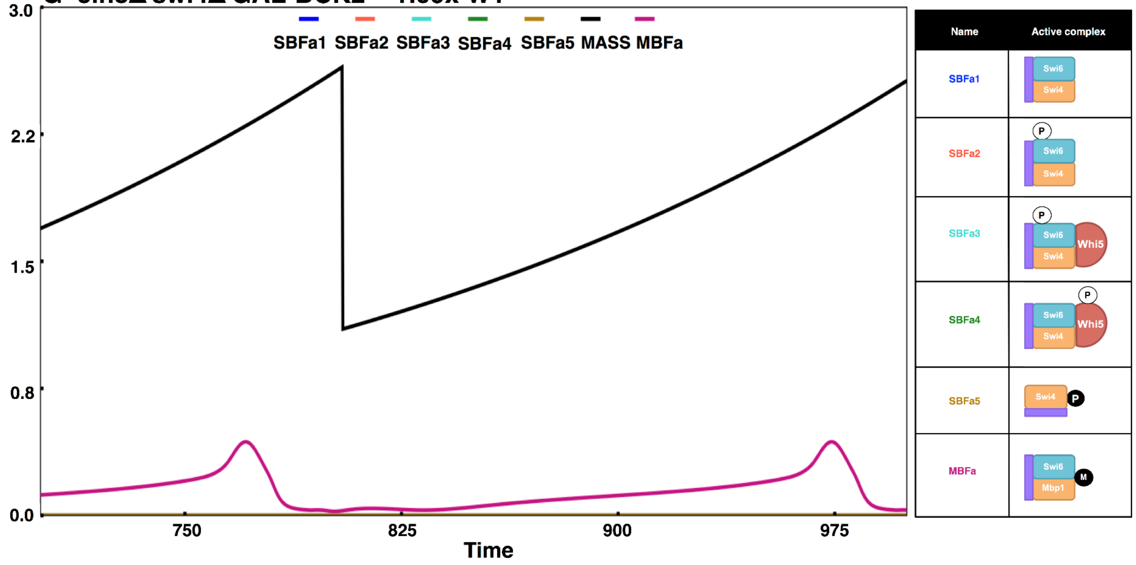


H mbp1Δ GAL-WHI5 – 1.13G

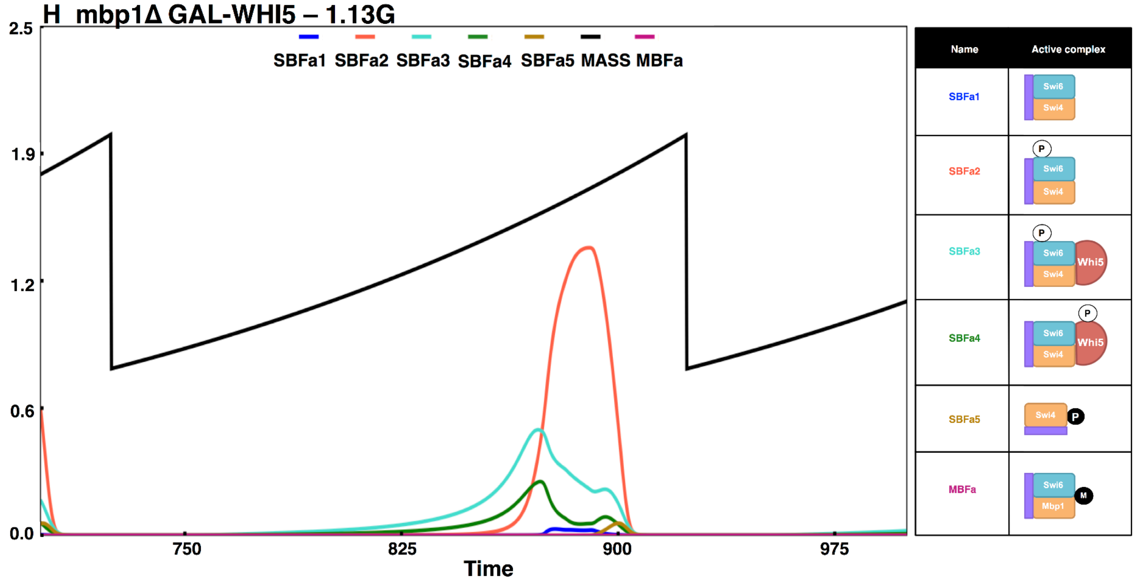

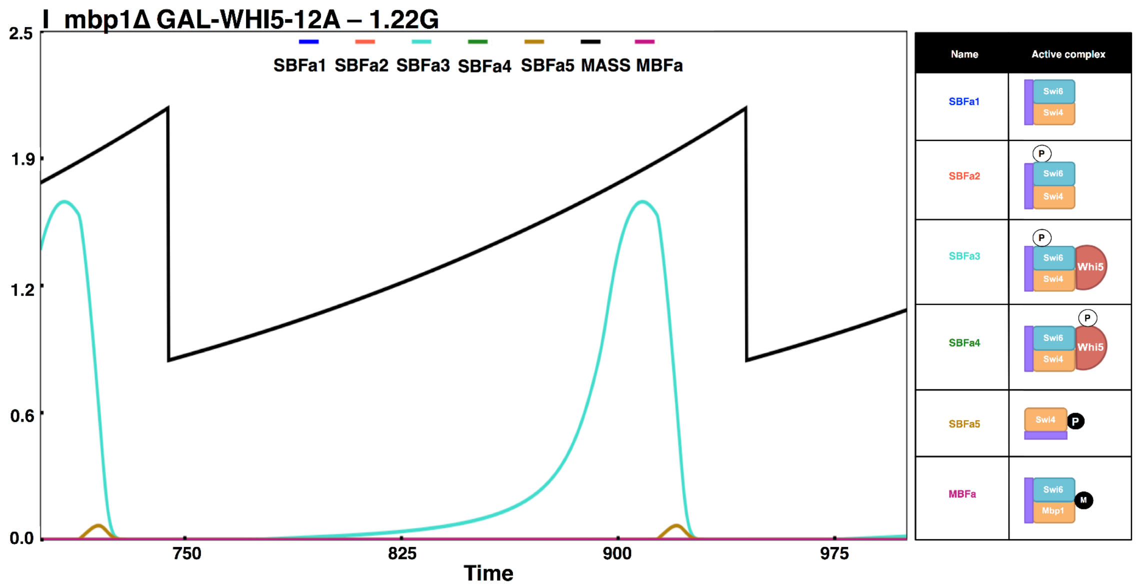


Figure S10. Few model predictions and validations.

(A) *bck2Δ mbp1Δ* (Cln-activated forms of SBF are present in varying fractions (SBFa2>SBFa3, SBFa4> SBFa1); cells are viable), (B) *bck2Δ mbp1Δ GAL-WHI5* (Cln-activated forms of SBF are present (SBFa2, SBFa3, SBFa4) even if they are lesser than in A; cells are still viable), (C) *cln3Δ mbp1Δ* (SBFa2 > SBFa3 >> SBFa4 > SBFa5; no MBF is present; cells are viable), (D) *cln3Δ mbp1Δ swi6Δ* (Swi4dimers (SBFa5) are present sufficiently enough to rescue cells; cells are viable), (E) *cln3Δ mbp1Δ whi5Δ* (Inhibition on SBF is relieved); cells are rescued), (F) *cln3Δ swi4Δ whi5Δ* (More Bck2-activated MBF is present (MBFa), there's no SBF; cells are viable), (G) *cln3Δ swi4Δ GAL-BCK2* (There's a sufficient amount of Bck2-activated MBF (MBFa); cells are rescued), (H) *mbp1Δ GAL-WHI5* cells are viable since Whi5 is predicted to get activated and is present in the forms of SBFa2, SBFa3, and SBFa4 (I) *mbp1Δ GAL-WHI5-12A* cells are viable because Whi5 get converted to the activated form SBFa3.
