# Supplementary material for: Modeling the START transition in the budding yeast cell cycle
Source: PLoS Comput Biol. 2024 Aug 2;20(8):e1012048. doi: 10.1371/journal.pcbi.1012048 (PMC11324117; doi:10.1371/journal.pcbi.1012048)
Supplement: S1 Table — (PDF) [file pcbi.1012048.s011.pdf]

Table S1. Functions of different cyclins in the budding yeast cell cycle

Cyclin	Function
Cln1,2	- Bud emergence, START<br>- Inhibiting kinase inhibitors Sic1, Cdc6 and Cdh1 (which inhibit Clbs)<br>- In the model, Cln2 represents Cln1,2
Cln3	- Activates SBF (TF for Cln1,2) and MBF (TF for Clb5,6) in response to cell size
Clb1,2	- Late mitotic events, including elongation of short mitotic spindle<br>- Promotes cell cycle transition into mitosis<br>- In the model, Clb2 represents Clb1,2
Clb3,4	- Early mitotic events, including the formation of short mitotic spindle<br>- Involved in DNA synthesis, spindle assembly as well as G2/M transition
Clb5,6	- DNA Synthesis<br>- Inhibiting kinase inhibitors Sic1, Cdc6 and Cdh1<br>- In the model, Clb5 represents Clb5,6
