# Supplementary material for: Modeling the START transition in the budding yeast cell cycle
Source: PLoS Comput Biol. 2024 Aug 2;20(8):e1012048. doi: 10.1371/journal.pcbi.1012048 (PMC11324117; doi:10.1371/journal.pcbi.1012048)
Supplement: S2 Table — (PDF) [file pcbi.1012048.s012.pdf]

Table S2. Description, abundance, regulation, and localization of START components in the model

Protein / Component	Description/Regulation
Whi5	***Description***<br>- Whi5 is a stoichiometric inhibitor of the transcription factor complexes, SBF [1,2] and MBF [1].<br>***Abundance***<br>- The abundance of Whi5 is roughly 1500 protein molecules per cell in an asynchronous culture [3]), although Whi5 is known to be transcriptionally regulated, showing a 3-fold variation, with the highest expression in S/G2/M phases [4,5]. Therefore, we assume available Whi5 (for inhibition) in G1 to be 1000 molecules.<br>- For simplicity, we assume total Whi5 to be conserved in the model.<br>***Regulation & Localization***<br>- Whi5 has 12 known CDK phosphorylation sites and 6 other non-CDK sites [6]. We assume Hill kinetics (with Hill's coefficient=5) for CDK phosphorylation of Whi5 to capture the non-linearity resulting from multi-site phosphorylation (compared and tested for a smaller version of model).<br>- We assume that Whi5 is phosphorylated by Cln3, Cln2, and Clb5 (with different efficiencies) and dephosphorylated by Cdc14 at mitotic exit [1,2,7]. We also assume that all forms of Whi5 (either free, or SBF-bound or SBF-promoter-bound) are subjected to phosphorylation and dephosphorylation.<br>- Whi5 is nuclear until late G1, and moves to cytoplasm prior to START, and stays cytoplasmic until mitotic exit [8]. This localization is dependent on phosphorylation of specific CDK phosphorylation sites on Whi5, and the transport protein Msn5.
Swi6	***Description***<br>- Swi6 is a **component** of the transcription factor, **SBF** and **MBF** [9,10].<br>***Abundance***<br>- It is 3 times more abundant than Whi5 [3].<br>- We assume total Swi6 to be conserved in the model.<br>***Regulation & Localization***<br>- Swi6 has 5 consensus CDK phosphorylation sites [11].<br>- We assume that the Cln kinase phosphorylation on Whi5 and its phosphorylation on Swi6 have the same dynamics. That is, we

	use the same Hill function to describe the phosphorylation of Cln kinase on Whi5 as well as on Swi6.<br>- In the model, the sites bearing the P-form – the activatory phosphorylations are done by Cln3, Cln2 and Clb5 with different efficiencies, and the dephosphorylation is by unspecified phosphatase.<br>- Phosphorylation at the S160 site, is known to be responsible for the cytoplasmic localization of Swi6 from mid-S phase until mitotic exit [12]. This is considered to be the Q-form (inactivated form) in the model and is required for cytoplasmic localization. The Q-form phosphorylation is carried out by Clbs (Clb5 and Clb2 in our model), and dephosphorylated by Cdc14 at mitotic exit [13]. Transport of Q-forms to the cytoplasm requires Msn5 and Swi4 [14].<br>- We assume phosphorylation (P- and Q-forms) occurs for all forms of Swi6 (either free or promoter-bound forms).
Swi4	***Description***<br>- Swi4 is a component of the transcription factor, SBF [9,10].<br>***Abundance***<br>- Based on observed protein abundances, we consider the relative abundance of Swi4 and Mbp1 to be ~0.55x as abundant as Whi5 [3]. Therefore, Swi4 is the limiting component of the SBF complex.<br>- We assume total Swi4 to be conserved in the model.<br>***Regulation and Localization***<br>- We assume that phosphorylation of Swi4 is necessary for SBF inactivation [15]. Phosphorylation of Swi4 subunit of SBF causes SBF to dissociate from the promoter.<br>- Swi4 is nuclear at all times [16]. In the model, phosphorylated Swi4 gets dephosphorylated by a constitutively active phosphatase, PP2A. The unphosphorylated form remains nuclear at all times.<br>- Since *swi6Δ* mutant is viable, but *swi6Δ bck2Δ* is inviable, we assume that Swi4 has some residual activity in the absence of SBF [9,17], but it requires Bck2 for its activity. The active Swi4 complex is also assumed to be inactivated by Clb2.
Mbp1	***Description***<br>- Mbp1 is a component of the transcription factor, MBF [9,10].<br>***Abundance***

	- As mentioned above, we assume Mbp1 to have a relative abundance of 0.55x w.r.t Whi5. Mbp1 is, therefore, a limiting component of MBF [3].<br>- We assume total Mbp1 to be conserved in the model.<br>***Regulation and Localization***<br>- MBF regulation is driven by Cln3 and Bck2 for activation [9], and Nrm [18] and Clb2 (to a much lesser extent) for inactivation.<br>- Since little information is available about the modification or localization of Mbp1, they are not considered in the model.
Msn5	***Description***<br>- Msn5 is a transport protein that exports phosphoproteins (Swi6 and Whi5 in our model) from the nucleus to the cytoplasm [7,14].<br>***Regulation***<br>- We do not consider the regulation of Msn5. It is assumed to be constant in our model.
Cln3 & Bck2 (Modification from Chen2004)	***Regulation & Localization***<br>- **Activation** of Cln3, which is by nuclear import, depends on chaperone protein, Ydj1, availability of which is proposed to serve as a sensor for cell growth rate [19]. In the model, Ydj1 depends on mass. We also assume that the abundance of Cln3 depends on mass. This assumption is necessary for mutant cells with longer G1 to have shorter G2/M so that the total cycle time (G1 + S/G2/M) is the same as wild type cells. Thus, mutant cells can maintain their size homeostasis generation after generation.<br>- **Inactivation** of Cln3 is done by Ssa1 [19] (detailed in Main text). To explain the change of Cln3 localization during the cell cycle, (being nuclear in late G1, just prior to START and becoming cytoplasmic from late S phase on), we assume that Ssa1 is activated by Clb2 and Swi5, both of which accumulate during M-phase.<br>- Currently, there is no evidence for Bck2 being regulated the same way as Cln3. But since Bck2 is known to be a cell size regulator too [20], we assume that its activation and inactivation depend on similar mechanisms that are contingent on mass and Clb2, Swi5 (instead of Ydj1 and Ssa1).
Promoters	- We assume the promoter concentration to be 0.2 relative to Whi5 abundance, corresponding to 200 SBF-regulated genes.<br>- We consider two species, Prom2 and Prom5, corresponding to genes regulated by SBF and MBF, respectively.

References

1. Costanzo M, Nishikawa JL, Tang X, Millman JS, Schub O, Breitkreuz K, et al. CDK Activity Antagonizes Whi5, an Inhibitor of G1/S Transcription in Yeast. Cell. 2004;117: 899–913. doi:10.1016/j.cell.2004.05.024
2. de Bruin RAM, McDonald WH, Kalashnikova TI, Yates J, Wittenberg C. Cln3 Activates G1-Specific Transcription via Phosphorylation of the SBF Bound Repressor Whi5. Cell. 2004;117: 887–898. doi:10.1016/j.cell.2004.05.025
3. Ghaemmaghami S, Huh W-K, Bower K, Howson RW, Belle A, Dephoure N, et al. Global analysis of protein expression in yeast. Nature. 2003;425: 737–741. doi:10.1038/nature02046
4. Pramila T. The Forkhead transcription factor Hcm1 regulates chromosome segregation genes and fills the S-phase gap in the transcriptional circuitry of the cell cycle. Genes Dev. 2006;20: 2266–2278. doi:10.1101/gad.1450606
5. Schmoller KM, Turner JJ, Kõivomägi M, Skotheim JM. Dilution of the cell cycle inhibitor Whi5 controls budding-yeast cell size. Nature. 2015;526: 268–272. doi:10.1038/nature14908
6. Wagner A, Grillitsch K, Leitner E, Daum G. Mobilization of steryl esters from lipid particles of the yeast Saccharomyces cerevisiae. Biochim Biophys Acta BBA - Mol Cell Biol Lipids. 2009;1791: 118–124. doi:10.1016/j.bbalip.2008.11.004
7. Taberner FJ, Quilis I, Igual JC. Spatial regulation of the Start repressor Whi5. Cell Cycle. 2009;8: 3013–3022. doi:10.4161/cc.8.18.9621
8. Talia SD, Skotheim JM, Bean JM, Siggia ED, Cross FR. The effects of molecular noise and size control on variability in the budding yeast cell cycle. Nature. 2007;448: 947–951. doi:10.1038/nature06072
9. Koch C, Moll T, Neuberg M, Ahorn H, Nasmyth K. A role for the transcription factors Mbp1 and Swi4 in progression from G1 to S phase. Science. 1993;261: 1551–1557. doi:10.1126/science.8372350
10. Moll T, Tebb G, Surana U, Robitsch H, Nasmyth K. The role of phosphorylation and the CDC28 protein kinase in cell cycle-regulated nuclear import of the S. cerevisiae transcription factor SWI5. Cell. 1991;66: 743–758. doi:10.1016/0092-8674(91)90118-i
11. Wijnen H, Landman A, Futcher B. The G 1 Cyclin Cln3 Promotes Cell Cycle Entry via the Transcription Factor Swi6. Mol Cell Biol. 2002;22: 4402–4418. doi:10.1128/MCB.22.12.4402-4418.2002
12. Sidorova JM, Mikesell GE, Breeden LL. Cell cycle-regulated phosphorylation of Swi6 controls its nuclear localization. Mol Biol Cell. 1995;6: 1641–1658. doi:10.1091/mbc.6.12.1641
13. Geymonat M, Spanos A, Wells GP, Smerdon SJ, Sedgwick SG. Clb6/Cdc28 and Cdc14 Regulate Phosphorylation Status and Cellular Localization of Swi6. Mol Cell Biol. 2004;24: 2277–2285. doi:10.1128/MCB.24.6.2277-2285.2004
14. Queralt E, Igual JC. Cell Cycle Activation of the Swi6p Transcription Factor Is Linked to Nucleocytoplasmic Shuttling. Mol Cell Biol. 2003;23: 3126–3140. doi:10.1128/MCB.23.9.3126-3140.2003
15. Siegmund RF, Nasmyth KA. The Saccharomyces cerevisiae Start-specific transcription factor Swi4 interacts through the ankyrin repeats with the mitotic Clb2/Cdc28 kinase and through its conserved carboxy terminus with Swi6. Mol Cell Biol. 1996;16: 2647–2655. doi:10.1128/MCB.16.6.2647
16. Baetz K, Andrews B. Regulation of Cell Cycle Transcription Factor Swi4 through Auto-Inhibition of DNA Binding. Mol Cell Biol. 1999;19: 6729–6741. doi:10.1128/MCB.19.10.6729
17. Dirick L, Nasmyth K. Positive feedback in the activation of G1 cyclins in yeast. Nature. 1991;351: 754–757. doi:10.1038/351754a0
18. de Bruin RAM, Kalashnikova TI, Aslanian A, Wohlschlegel J, Chahwan C, Yates JR, et al. DNA replication checkpoint promotes G1-S transcription by inactivating the MBF repressor Nrm1. Proc Natl Acad Sci. 2008;105: 11230–11235. doi:10.1073/pnas.0801106105
19. Vergés E, Colomina N, Garí E, Gallego C, Aldea M. Cyclin Cln3 Is Retained at the ER and Released by the J Chaperone Ydj1 in Late G1 to Trigger Cell Cycle Entry. Mol Cell. 2007;26: 649–662. doi:10.1016/j.molcel.2007.04.023
20. Wijnen H, Futcher B. Genetic Analysis of the Shared Role of CLN3 and BCK2 at the G1-S Transition in Saccharomyces cerevisiae. Genetics. 1999;153: 1131–1143. doi:10.1093/genetics/153.3.1131
