# Supplementary material for: Modeling the START transition in the budding yeast cell cycle
Source: PLoS Comput Biol. 2024 Aug 2;20(8):e1012048. doi: 10.1371/journal.pcbi.1012048 (PMC11324117; doi:10.1371/journal.pcbi.1012048)
Supplement: S3 Table — (PDF) [file pcbi.1012048.s013.pdf]

Table S3. List of Key Assumptions

SBF and MBF overlap functionally.
START proteins Swi4, Swi6, Mbp1 and Whi5 are expressed constitutively.
Whi5 inhibits SBF strongly and MBF weakly.
Relative abundances of Swi4, Mbp1, Swi6, Whi5, SCB, MCB are 5.5, 5.5, 30, 10, 2, 2.
Cln3-kinase activates SBF in two ways: by activating Swi6 and by inactivating Whi5.
Phosphorylations of SBF by G1/S cyclins are described by a Hill function with nH = 5.
Clb1,2-kinases inactivate SBF by phosphorylating Swi4 and Swi6.
Whi5 is exported from the nucleus by Msn5 when it is phosphorylated.
Phosphorylated Whi5 dissociates from the promoter if the Swi6 moiety in the Swi4/Swi6/Whi5 complex is also phosphorylated.
Swi4/Swi6 complex is exported from the nucleus by Msn5 when Swi6 is phosphorylated.
Swi4 is dephosphorylated constitutively by an active phosphatase and, hence, it is nuclear throughout the cell cycle.
Bck2 and Cln3 activate SBF and MBF in a similar manner.
A Swi4-only form of SBF (that requires Swi4 and Bck2 but not Swi6) is responsible for the viability of swi6Δ cells.
MBF, like SBF, is activated by Cln3 and Bck2; it is inactivated by Clb2 and Nrm1.
Nuclear entry of Cln3 and Bck2 are controlled by Ydj1 and Ssa1 in a cell-size dependent manner.
Ssa1 is responsible for nutrient modulation of the critical size threshold for START.
Rates of Clb1,2 and Clb5,6 synthesis increase with cell mass.
The various promoter-bound forms SBF and MBF have different activities.
A simulated cell is considered viable if and only if it satisfies certain rules for “viability”.
