# Supplementary material for: Modeling the START transition in the budding yeast cell cycle
Source: PLoS Comput Biol. 2024 Aug 2;20(8):e1012048. doi: 10.1371/journal.pcbi.1012048 (PMC11324117; doi:10.1371/journal.pcbi.1012048)
Supplement: S4 Table — (PDF) [file pcbi.1012048.s014.pdf]

Table S4.Modifications in Parameter & Initial conditions corresponding to mutants. (Mutants exclusive to the current model are emphasized in bold).

Mutants	Parameters changed in model
**Loss of function mutants**	
**G1, S**	
cln3Δ	CLN3T=0
bck2Δ	BCK2T=0
whi5Δ	init WHI5=0
WHI5-12A	ef5p=0, mdt=150, init WHI5 = whi5op*WHI5
swi4Δ	init SWI4=0
swi6Δ	init SWI6=0
SWI6-SA4	ef6p=ef6q=0
mbp1Δ	init MBP1=0
msn5Δ	MSN5=0
cln2Δ	ksn2'=ksn2"=ksn2'''=0; init CLN2=0
clb5Δ	ksb5'=ksb5"=ksb5'''=0; init CLB5=0
CLB5-dbΔ	kdb5"=0
**Cyclin Antagonists**	
sic1Δ	ksc1'=ksc1"=0; init SIC1=SIC1P=C2=C2P=C5=C5P=0
cdc6Δ (cdc6 2-49Δ)	ksc6'=ksc6"=ksc6'''=0; init CDC61=CDC6P=F2=F2P=F5=F5P=0
ckiΔ	ksc1'=ksc1"=ksc6'=ksc6"=ksc6'''=0;<br>init SIC1=SIC1P=C2=C2P=C5=C5P=CDC61=CDC6P=F2=F2P=F5=F5P
cdh1Δ	kscdh=0; CDH1=CDH1i=0
**M-phase**	
swi5Δ	ksc1"=ksf6"=0
clb2Δ	ksb2'=ksb2"=0
CLB2-dbΔ	kdb2"=0.25*kdb2", kdb2'''=0
CLB1 clb2Δ	ksb2'=0.33*ksb2', ksb2"=0.33*ksb2"
cdc20Δ	ks20'=ks20"=0
cdc20-ts	
apc-ts	ks20'=ks20"=kscdh=0; init CDH1=CDH1i=0
APC-A	ka20"=0
pds1Δ	kspds'=0; PDS1=PE=ESP1=0
PDS1-dbΔ	kdpds"=kdpds'''=0
esp1-ts	kasesp=0.002*kasesp, kdirent=0.002*kdirent, ki=0.04*ki
ppxΔ	PP2AT=0
tem1-ts	ka15"=0.003*ka15
net1-ts	kasrent=0.04*kasrent, kasrentp=0.04*kasrentp
cdc15Δ	kpnet"=0
TAB6-1	kasrent=0.02*kasrent, kasrentp=0.02*kasrentp
cdc14-ts	ks14=0; init CDC14=0
mad2Δ	mad2h=0.01; init MAD2=0.01
bub2Δ	bub2h=bub2l=0; init BUB2=0
Cells in nocodazole	ksspn=0

Over-expression (gain of function) mutants	
Cells in galactose	mdt=150 (applicable for all *GAL* mutants below)
GAL-CLN3	CLN3T=kgalcln3*CLN3T
GAL-BCK2	BCK2=3*BCK2T
GAL-WHI5	init WHI5=10*WHI5
GAL-WHI5-12A	ef5p=0; init WHI5=10*WHI5
GAL-CLN2	ksn2'=0.165
GAL-CLB5	ksb5'=0.016
GAL-SIC1	ksc1'=0.132
GAL-CDC6	ksf6'=0.4
GAL-CLB2	ksb2'=0.38
GAL-CDC20	ks20'=6
GALL-CDC20	ks20'=0.6
GAL-ESP1	init ESP1=4*ESP1, PE=4*PE
GAL-PDS1	kspds'=0.2
GAL-PPX	PP2AT=6
GAL-CDC15	init CDC15i=20*CDC15i, CDC15=20*CDC15 (20 copies)
GAL-NET1	ksnet=4*ksnet
GAL-CDC14	ks14=4*ks14
GAL-TEM1	init TEM1GDP=20*TEM1GDP, TEM1GTB=20*TEM1GTB (20 copies)
CLN3-1	CLN3T=kmccln3*CLN3T
CDH1 constitutively active	mdt=150, kicdh''=0, kscdh=3*kscdh

GAL-SIC-dbΔ	mdt=150, ksc1'=kgalsic1 , kd3c1=0.132 (same as GAL-SIC1)
GAL-CLB5-dbΔ	mdt=150, ksb5'=kgalblb5 , kdb5''=0.016 (same as GAL-CLB5)
GAL-PDS1-dbΔ	kdpds''=kdps'''=0
**Multi-copy (mc) mutants**	
mc BCK2	BCK2T=5*BCK2T
mc CLN2	ksn2''=4*ksn2'', ksn2'''=4*ksn2'''
mc CLB5	ksb5'=4*ksb5', ksb5''=4*ksb5'', ksb5'''=4*ksb5'''
mc GAL-CLB2	mdt=150, ksab2'=0.72
mc SIC1	ksc1'=4*ksc1', ksc1''=4*ksc1''
mc CDC6	ksf6'=0.4*ksf6', ksf6''=0.4*ksf6'', ksf6'''=0.4*ksf6'''
mc CDC20	ks20'=5*ks20', ks20''=5*ks20''
mc CDC14	ks14=3*ks14
mc TEM1	init TEM1GDP=20*TEM1GDP, TEM1GTB=20*TEM1GTB (20 copies)
mc CDC15	init CDC15i=20*CDC15i, CDC15=20*CDC15 (20 copies)
