# Supplementary material for: Modeling the START transition in the budding yeast cell cycle
Source: PLoS Comput Biol. 2024 Aug 2;20(8):e1012048. doi: 10.1371/journal.pcbi.1012048 (PMC11324117; doi:10.1371/journal.pcbi.1012048)
Supplement: S5 Table — (PDF) [file pcbi.1012048.s015.pdf]

S5 Table. START mutants

In blue: Predictions; In red: Contradictions; *GAL* mutants are scaled w.r.t *GAL*-WT.
Size of WT in Glucose: 1x; Size of WT in Galactose: 1G

#	Genotype	Experimental phenotypes	Simulation results	References
Mutants pertaining to SBF, MBF; G1, G1/S, S cyclins; Bck2 and cyclin antagonists.				
1	*WHI5-12A*	1x	1.07x	Wagner 09 [1]
2	*SWI6-SA4*	1x	1.09x	Wagner 09 [1]
3	*SWI6-SA4 WHI5-12A*	1.4x	1.73x	Wagner 09 [1]
4	*GAL-WHI5-12A*	>1x	1.2G	Wagner 09 [1]
5	*GAL-WHI5-12A SWI6-SA4*	Inviable	G1 arrest	Wagner 09 [1]
6	*bck2Δ*	1.3x	1.32x	Wijnen 99 [2]
7	*Multi-copy BCK2*	0.8x	0.77x	Di Como 95 [3]
8	*GAL-BCK2*	Viable	0.75G	Costanzo 04 [4]
9	*cln2Δ*	3.2x	1.92x	Dirick 95 [5]
10	*GAL-CLN2*	0.5x	0.52G	Dirick 95 [5]
11	*Multi-copy CLN2*	Viable, <1x	0.81x	–
12	*cln3Δ*	1.8-2.7x	2.2x	Dirick 95, Costanzo 04 [4,5]
13	*CLN3-1*	0.7x	0.49x	Costanzo 04 [4]
14	*GAL-CLN3*	0.5x	0.40G	Tyers 92 [6]
15	*whi5Δ*	0.6-0.7x	0.82x	de Bruin 04, Costanzo 04 [4,7]
16	*GAL-WHI5*	>1x	1.09G	de Bruin 04, Costanzo 04 [4,7]
17	*clb5,6Δ*	>1x	1.11x	Schwob 93 [8]
18	*Multi-copy CLB5*	CEN, Viable	0.98x, 4 copies	–
19	*GAL-CLB5*	Viable	0.95G	Schwob 93 [8]
20	*CLB5-dbΔ*	Viable	1.04x	Wasch 02 [9]
21	*GAL-CLB5dbΔ*	Inviable, DNA synthesis not advanced	T arrest, ORI advanced by 7.8’	Schwob 94 [8]

22	*triple-cln*	Inviable	G1 arrest ORI 248’	Richardson 89 [10]
23	*mbp1Δ*	1.3x	1.15x	Ferrezuelo 09, Koch 93 [11,12]
24	*swi4Δ*	1.3-1.5x	1.42x	Wijnen 99, Wijnen 02 [2,13]
25	*swi6Δ*	2.4x	2.25x	Wijnen 02 [13]
26	*msn5Δ*	1.4x	1.34x	Queralt 03 [14]
27	*sic1Δ*	<1x	0.90x	Schneider 96 [15]
28	*GAL-SIC1*	>1x	1.09G	Nugro 94, Verma 97 [16,17]
29	*GAL-SIC1dbΔ*	G1 arrest	G1 arrest	Verma 97 [17]
30	*Multi-copy SIC1*	Viable	1.10x	–
31	*cdc6Δ*	<1x	1.02x	Calzada 01 [18]
32	*GAL-CDC6*	Viable	1.06G	Archambault 03 [19]
33	*Multi-copy CDC6*	Viable	1.02x	–
34	*ckiΔ*	Viable, <1x	0.88x	Wasch 02 [9]
35	*swi5Δ*	Viable	0.98x	Toyn 97, Giaever 02 [20,21]
36	*cdh1Δ*	Viable, <1x	0.94x	Schwab 97, Wasch 02 [8,9]
37	*CDH1 const active*	G2 arrest	G2 arrest	Zachariae 99 [22]
38	*bck2Δ cln2Δ*	>>1x	2.44x	Epstein 94 [23]
39	*bck2Δ cln3Δ*	Inviable	G1 arrest	Wijnen 99 [2]
40	*bck2Δ cln3Δ whi5Δ*	1.4x	1.43x	de Bruin 04, Costanzo 04 [4,7]
41	*bck2Δ cln3Δ whi5Δ mbp1Δ*	1.4x	1.43x	de Bruin 04 [7]
42	*bck2Δ cln3Δ whi5Δ swi4Δ*	Inviable	G1 arrest	de Bruin 04 [7]
43	*bck2Δ cln3Δ GAL-CLN2*	Rescued	0.39G	di Como 95 [3]
44	*bck2Δ cln3Δ sic1Δ*	Inviable	G1 arrest, ORI 254’	Wijnen 99 [2]
45	*bck2Δ mbp1Δ*	Prediction: Viable	1.43x	–

46	*bck2Δ mbp1Δ GAL-WHI5*	Prediction: Viable	1.55G	–
47	*bck2Δ swi4Δ*	1.55x	1.71x	Wijnen 99 [2]
48	*bck2Δ swi6Δ*	Inviable	G1 arrest	Wijnen 02 [13]
49	*bck2Δ swi6Δ SWI6-SA4*	Rescued	1.56x	Wijnen 02 [13]
50	*bck2Δ swi6Δ GAL-CLB5*	Prediction: Rescued	1.89G	–
51	*bck2Δ swi6Δ GAL-CLN3*	Prediction: Inviable	Inviable	–
52	*bck2Δ swi6Δ GAL-CLN2*	Prediction: Rescued	1.35G	–
53	*bck2Δ swi6Δ whi5Δ*	Inviable	G1 arrest	de Bruin 04 [7]
54	*bck2Δ whi5Δ*	0.85x	0.91x	de Bruin 04, Costanzo 04 [4,7]
55	*bck2Δ GAL-WHI5*	Viable, >*GAL-WHI5*	1.49G	Costanzo 04 [4]
56	*bck2Δ GAL-WHI5-12A*	Prediction: Viable, large	1.72G	–
57	*GAL-BCK2 whi5Δ*	0.5x	0.57G	Costanzo 04 [4]
58	*cln1Δ cln2Δ clb5Δ clb6Δ*	Inviable	G1 arrest	Schwob 93 [8]
59	*cln1Δ cln2Δ cdh1Δ*	Viable	T arrest	Cross 02 [24]
60	*cln1Δ cln2Δ GAL-CLN2 cdh1Δ*	Viable	0.11G	Cross 02 [24]
61	*cln1Δ cln2Δ sic1Δ*	Viable	1.08x	Dirick 95 [5]
62	*cln1Δ cln2Δ GAL-SIC1*	G1 arrest	G1 arrest	Cross 02 [24]
63	*GAL-CLN2 GAL-SIC1*	Viable	2.85G	Cross 02 [24]
64	*GAL-CLN2 cdh1Δ GAL-SIC1*	Viable	0.52G	Cross 02 [24]
65	*cln2Δ cdh1Δ GAL-SIC1*	Inviable	G1 arrest	Cross 02 [24]
66	*cln3Δ mbp1Δ*	Prediction: Inviable	2.25x	–
67	*cln3Δ mbp1Δ swi6Δ*	Prediction: Rescued, ~*swi6Δ*	1.59x	–
68	*cln3Δ mbp1Δ whi5Δ*	Prediction: Rescued	1.23x	–
69	*cln3Δ mbp1Δ mc-BCK2*	Prediction: Viable	0.98x	–
70	*cln3Δ mbp1Δwhi5Δ bck2Δ*	Prediction: Viable	1.43x	–
71	*cln3Δ swi4Δ*	Inviable	Inviable	Ferrezuelo 09 [11]
72	*cln3Δ swi4Δ whi5Δ*	Prediction: Rescued	2.36x	–
73	*cln3Δ swi4Δ mc-BCK2*	Prediction: Viable	1.30x	–
74	*cln3Δ swi4Δ whi5Δ sic1Δ*	Prediction: Rescued	1.73x	–

75	*cln3Δ swi4Δ GAL-BCK2*	Prediction: Rescued	1.50G	–
76	*cln3Δ swi4Δ whi5Δ GAL-BCK2*	Prediction: Rescued	0.83x	–
77	*cln3Δ swi6Δ*	2.4x, ~*swi6Δ*	2.34x	Wijnen 02 [13]
78	*CLN3-1 swi6Δ*	2.4x, ~*swi6Δ*	2.29x	Wijnen 02 [13]
79	*cln3Δ whi5Δ*	0.7-0.9x	1.07x	de Bruin 04, Costanzo 04 [4,7]
80	*CLN3-1 whi5Δ*	Viable, small	0.36G	Costanzo 04 [4]
81	*cln3Δ GAL-WHI5*	Inviable	2.76G	Costanzo 04, Wagner 09 [1,4]
82	*cln3Δ GAL-WHI5-12A*	Inviable	2.79G	Costanzo 04 [4]
83	*triple-cln GAL-CLN2*	Viable	0.93G	Cross 91 [25]
84	*triple-cln GAL-CLN3*	Viable	1.36G	Cross 90 [26]
85	*triple-cln sic1Δ*	>>1x	1.84x	Tyers 96 [27]
86	*triple-cln cdh1Δ*	T arrest	T arrest	Schwab 97 [28]
87	*triple-cln mc-CLB5*	CEN, Viable	2.58x	Epstein 92 [29]
88	*triple-cln GAL-CLB5*	Viable	1.81G	Schwob 93 [8]
89	*triple-cln mc-BCK2*	Viable	1.14x	Epstein 94 [23]
90	*triple-cln GAL-CLB2*	G1 arrest	T arrest, ORI 148’	Amon 94 [30]
91	*mbp1Δ swi4Δ*	Inviable	G1 arrest	Koch 93 [12]
92	*mbp1Δ whi5Δ*	<1x	0.92x	de Bruin 04 [4]
93	*mbp1Δ GAL-WHI5*	Prediction: Viable	1.13G	–
94	*mbp1Δ GAL-WHI5-12A*	Prediction: Viable	1.22G	–
95	*mbp1Δ swi6Δ*	2.4x, ~*swi6Δ*	1.59x	Ferrezulo 09 [11]
96	*swi4Δ swi6Δ*	Inviable	G1 arrest	Dirick 91 [31]
97	*swi4Δ swi6Δ SWI6-SA4*	Rescued	1.38x	Wijnen 02 [13]
98	*swi4Δ swi6Δ GAL-CLB5*	Prediction: Viable, >1x	1.87G	–
99	*swi4Δ swi6Δ GAL-CLN3*	Inviable	Inviable	–
100	*swi4Δ swi6Δ GAL-CLN2*	Rescued	1.30G	–
101	*swi4Δ whi5Δ*	1.3-1.5, ~*swi4Δ*	1.19x	Jorgensen 02 [32]
102	*swi4Δ GAL-WHI5*	Viable, ~*swi4Δ*	1.82G	de Bruin 04 [4]
103	*swi4Δ swi6Δ whi5Δ*	Prediction: Inviable	Inviable	–

104	*swi6Δ whi5Δ*	2.4x, ~*swi6Δ*	1.59x	Costanzo 04 [4]
105	*swi6Δ GAL-WHI5*	Inviable	1.07G	Costanzo 04 [4]
106	*swi6Δ mc-WHI5*	Prediction: Viable	1.60x	–
107	*msn5Δ swi4Δ*	Inviable	1.79x	Queralt 06 [33]
108	*msn5Δ swi6Δ*	Inviable	1.59x	Queralt 06 [33]
109	*cdh1Δ sic1Δ*	Inviable	T arrest	Schwab 97, Archambault 03 [19,28]
110	*cdh1Δ cdc6Δ*	Viable, >1x	0.96x	Calzada 01 [18]
111	*cdh1Δ sic1Δ cdc6Δ*	Inviable	T arrest	Archambault 03 [19]
112	*cdh1Δ swi5Δ*	Inviable	T arrest	Archambault 03 [19]
113	*cdh1Δ swi5Δ GAL-SIC1*	Rescued	1.06G	Archambault 03 [19]
114	*GAL-CLB5 cdh1Δ*	Inviable*; Viable in model	0.83G	Chen 04 [34]
115	*GAL-CLB5 sic1Δ*	Inviable	Inviable, ORI not relicensed	Jacobson 00 [35]
116	*CLB5-dbΔ sic1Δ*	Inviable, ORI not relicensed	Inviable, ORI not relicensed	Jacobson 00, Wasch 02 [9,35]
Some essential exit mutants				
117	*triple-cln apc-ts*	M arrest	M arrest	Irniger 97 [36]
118	*cdh1Δ sic1Δ GALL-CDC20*	Viable, >1x	0.97G	Cross 03 [37]
119	*cdh1Δ sic1Δ cdc6Δ GALL-CDC20*	Rescued	1.01G	Cross 03 [37]
120	*CLB5-dbΔ pds1 Δ*	Viable	1.15x	Wasch 02 [9]
121	*CLB5-dbΔ pds1Δ cdc20Δ*	T arrest	T arrest	Wasch 02 [9]
122	*cdc20Δ*	Inviable	M arrest	Lim 98 [38]
123	*pds1Δ*	Viable	1.08x	Yamamoto 96 [39]
124	*tem1-ts*	Inviable	T arrest	Shirayama 94 [40]
125	*cdc15Δ*	Inviable	T arrest	Jasperson 00 [35]
126	*net1-ts*	Viable, >1x	1.08x	Visintin 99 [41]

127	*cdc14Δ*	Inviable	T arrest	Visintin 98 [42]
128	*ppxΔ*	Viable	1.27x	Wang 97 [43]
129	*cdc20Δ clb5Δ*	M arrest	M arrest	Shirayama 98 [44]
130	*cdc20Δ pds1Δ*	T arrest	T arrest	Shirayama 98 [44]
131	*cdc20Δ clb5Δ pds1Δ*	Viable, >1x	1.39x	Shirayama 98 [44]
132	*cdc20Δ GAL-ESP1*	T arrest, Cdc14 out	T arrest, Cdc14 out	Uhlmann 99 [45]
133	*APC-A*	Viable	1.20x	Rudner 00, Cross 03 [37,46]
134	*APC-A cdh1Δ*	T arrest	T arrest	Cross 03 [37]
135	*APC-A cdh1Δ in GAL*	T arrest	T arrest	Cross 03 [37]
136	*APC-A cdh1Δ mc-SIC1*	Rescued	1.41x	Cross 03 [37]
137	*APC-A cdh1Δ GAL-SIC1*	Rescued	1.14G	Cross 03 [37]
138	*APC-A cdh1Δ mc-CDC6*	Rescued	1.16x	Cross 03 [37]
139	*APC-A cdh1Δ GAL-CDC6*	Rescued	1.11G	Cross 03 [37]
140	*APC-A cdh1Δ mc-CDC20*	Rescued	0.84x	Cross 03 [37]
141	*APC-A sic1Δ*	Rescued	1.06x	Cross 03 [37]
142	*APC-A GAL-CLB2*	T arrest	T arrest	Cross 03 [37]

* Fred Cross: Although *GAL-CLB5 cdh1Δ* is ultimately inviable or extremely slow-growing on galactose, these mutants are not associated with any obvious problems in a short-term experiment. These cells go through several doublings, probably without much difficulty, on galactose medium, and they remain reasonably viable when returned to glucose, like *GAL-CLB5* cells. So, it is not reasonable to expect the mathematical model to predict the inviability of *GAL-CLB5 cdh1Δ* mutants. They should probably look viable.

References | for S5 Table

1. Wagner A, Grillitsch K, Leitner E, Daum G. Mobilization of steryl esters from lipid particles of the yeast Saccharomyces cerevisiae. Biochim Biophys Acta BBA - Mol Cell Biol Lipids. 2009;1791: 118–124. doi:10.1016/j.bbalip.2008.11.004
2. Wijnen H, Futcher B. Genetic Analysis of the Shared Role of CLN3 and BCK2 at the G1-S Transition in Saccharomyces cerevisiae. Genetics. 1999;153: 1131–1143. doi:10.1093/genetics/153.3.1131
3. Di Como CJ, Chang H, Arndt KT. Activation of *CLN1* and *CLN2* G_1 Cyclin Gene Expression by BCK2. Mol Cell Biol. 1995;15: 1835–1846. doi:10.1128/MCB.15.4.1835
4. Costanzo M, Nishikawa JL, Tang X, Millman JS, Schub O, Breitkreuz K, et al. CDK Activity Antagonizes Whi5, an Inhibitor of G1/S Transcription in Yeast. Cell. 2004;117: 899–913. doi:10.1016/j.cell.2004.05.024
5. Dirick L, Böhm T, Nasmyth K. Roles and regulation of Cln-Cdc28 kinases at the start of the cell cycle of Saccharomyces cerevisiae. EMBO J. 1995;14: 4803–4813. doi:10.1002/j.1460-2075.1995.tb00162.x
6. Tyers M, Tokiwa G, Futcher B. Comparison of the Saccharomyces cerevisiae G1 cyclins: Cln3 may be an upstream activator of Cln1, Cln2 and other cyclins. EMBO J. 1993;12: 1955–1968.

doi:10.1002/j.1460-2075.1993.tb05845.x
7. de Bruin RAM, McDonald WH, Kalashnikova TI, Yates J, Wittenberg C. Cln3 Activates G1-Specific Transcription via Phosphorylation of the SBF Bound Repressor Whi5. Cell. 2004;117: 887–898. doi:10.1016/j.cell.2004.05.025
8. Schwob E, Nasmyth K. CLB5 and CLB6, a new pair of B cyclins involved in DNA replication in Saccharomyces cerevisiae. Genes Dev. 1993;7: 1160–1175. doi:10.1101/gad.7.7a.1160
9. Wäsch R, Cross FR. APC-dependent proteolysis of the mitotic cyclin Clb2 is essential for mitotic exit. Nature. 2002;418: 556–562. doi:10.1038/nature00856
10. Richardson HE, Wittenberg C, Cross F, Reed SI. An essential G1 function for cyclin-like proteins in yeast. Cell. 1989;59: 1127–1133. doi:10.1016/0092-8674(89)90768-X
11. Ferrezuelo F, Aldea M, Futcher B. Bck2 is a phase-independent activator of cell cycle-regulated genes in yeast. Cell Cycle. 2009;8: 239–252. doi:10.4161/cc.8.2.7543
12. Koch C, Moll T, Neuberg M, Ahorn H, Nasmyth K. A role for the transcription factors Mbp1 and Swi4 in progression from G1 to S phase. Science. 1993;261: 1551–1557. doi:10.1126/science.8372350
13. Wijnen H, Landman A, Futcher B. The G_1 Cyclin Cln3 Promotes Cell Cycle Entry via the Transcription Factor Swi6. Mol Cell Biol. 2002;22: 4402–4418. doi:10.1128/MCB.22.12.4402-4418.2002
14. Queralt E, Igual JC. Cell Cycle Activation of the Swi6p Transcription Factor Is Linked to Nucleocytoplasmic Shuttling. Mol Cell Biol. 2003;23: 3126–3140. doi:10.1128/MCB.23.9.3126-3140.2003
15. Schneider BL, Yang Q-H, Futcher AB. Linkage of Replication to Start by the Cdk Inhibitor Sic1. Science. 1996;272: 560–562. doi:10.1126/science.272.5261.560
16. Nugroho TT, Mendenhall MD. An inhibitor of yeast cyclin-dependent protein kinase plays an important role in ensuring the genomic integrity of daughter cells. Mol Cell Biol. 1994;14: 3320–3328. doi:10.1128/mcb.14.5.3320-3328.1994
17. Verma R, Feldman RM, Deshaies RJ. SIC1 is ubiquitinated in vitro by a pathway that requires CDC4, CDC34, and cyclin/CDK activities. Mol Biol Cell. 1997;8: 1427–1437. doi:10.1091/mbc.8.8.1427
18. Calzada A, Sacristán M, Sánchez E, Bueno A. Cdc6 cooperates with Sic1 and Hct1 to inactivate mitotic cyclin-dependent kinases. Nature. 2001;412: 355–358. doi:10.1038/35085610
19. Archambault V, Li CX, Tackett AJ, Wasch R, Chait BT, Rout MP, et al. Genetic and biochemical evaluation of the importance of Cdc6 in regulating mitotic exit. Mol Biol Cell. 2003;14: 4592–4604. doi:10.1091/mbc.e03-06-0384
20. Toyn JH, Johnson AL, Donovan JD, Toone WM, Johnston LH. The Swi5 Transcription Factor of *Saccharomyces cerevisiae* Has a Role in Exit From Mitosis Through Induction of the cdk-Inhibitor Sic1 in Telophase. Genetics. 1997;145: 85–96. doi:10.1093/genetics/145.1.85
21. Giaever G, Chu AM, Ni L, Connelly C, Riles L, Véronneau S, et al. Functional profiling of the Saccharomyces cerevisiae genome. Nature. 2002;418: 387–391. doi:10.1038/nature00935
22. Zachariae W, Nasmyth K. Whose end is destruction: cell division and the anaphase-promoting complex. Genes Dev. 1999;13: 2039–2058. doi:10.1101/gad.13.16.2039
23. Epstein CB, Cross FR. Genes that can bypass the CLN requirement for Saccharomyces cerevisiae cell cycle START. Mol Cell Biol. 1994;14: 2041–2047. doi:10.1128/mcb.14.3.2041-2047.1994
24. Cross FR, Archambault V, Miller M, Klovstad M. Testing a Mathematical Model of the Yeast Cell Cycle. Solomon MJ, editor. Mol Biol Cell. 2002;13: 52–70. doi:10.1091/mbc.01-05-0265
25. Cross FR, Tinkelenberg AH. A potential positive feedback loop controlling CLN1 and CLN2 gene expression at the start of the yeast cell cycle. Cell. 1991;65: 875–883. doi:10.1016/0092-8674(91)90394-E
26. Cross FR. Cell Cycle Arrest Caused by *CLN* Gene Deficiency in *Saccharomyces cerevisiae* Resembles START-I Arrest and Is Independent of the Mating-Pheromone Signalling Pathway. Mol Cell Biol. 1990;10: 6482–6490. doi:10.1128/mcb.10.12.6482-6490.1990
27. Tyers M. The cyclin-dependent kinase inhibitor p40SIC1 imposes the requirement for Cln G1 cyclin function at Start. Proc Natl Acad Sci U S A. 1996;93: 7772–7776. doi:10.1073/pnas.93.15.7772
28. Schwab M, Lutum AS, Seufert W. Yeast Hct1 Is a Regulator of Clb2 Cyclin Proteolysis. Cell. 1997;90: 683–693. doi:10.1016/S0092-8674(00)80529-2
29. Epstein CB, Cross FR. CLB5: a novel B cyclin from budding yeast with a role in S phase. Genes Dev. 1992;6: 1695–1706. doi:10.1101/gad.6.9.1695
30. Amon A. Closing the cell cycle circle in yeast: G2 cyclin proteolysis initiated at mitosis persists until the activation of G1 cyclins in the next cycle. Cell. 1994;77: 1037–1050. doi:10.1016/0092-8674(94)90443-X
31. Dirick L, Nasmyth K. Positive feedback in the activation of G1 cyclins in yeast. Nature. 1991;351: 754–757. doi:10.1038/351754a0
32. Jorgensen P, Nishikawa JL, Breitkreutz B-J, Tyers M. Systematic Identification of Pathways That Couple Cell

Growth and Division in Yeast. Science. 2002;297: 395–400. doi:10.1126/science.1070850
33. Queralt E, Lehane C, Novak B, Uhlmann F. Downregulation of PP2ACdc55 Phosphatase by Separase Initiates Mitotic Exit in Budding Yeast. Cell. 2006;125: 719–732. doi:10.1016/j.cell.2006.03.038
34. Chen KC, Calzone L, Csikasz-Nagy A, Cross FR, Novak B, Tyson JJ. Integrative Analysis of Cell Cycle Control in Budding Yeast. Mol Biol Cell. 2004;15: 3841–3862. doi:10.1091/mbc.e03-11-0794
35. Jacobson MD, Gray S, Yuste-Rojas M, Cross FR. Testing Cyclin Specificity in the Exit from Mitosis. Mol Cell Biol. 2000;20: 4483–4493. doi:10.1128/MCB.20.13.4483-4493.2000
36. Irniger S, Nasmyth K. The anaphase-promoting complex is required in G1 arrested yeast cells to inhibit B-type cyclin accumulation and to prevent uncontrolled entry into S-phase. J Cell Sci. 1997;110: 1523–1531. doi:10.1242/jcs.110.13.1523
37. Cross FR. Two Redundant Oscillatory Mechanisms in the Yeast Cell Cycle. Dev Cell. 2003;4: 741–752. doi:10.1016/S1534-5807(03)00119-9
38. Lim HH, Goh P-Y, Surana U. Cdc20 is essential for the cyclosome-mediated proteolysis of both Pds1 and Clb2 during M phase in budding yeast. Curr Biol. 1998;8: 231–237. doi:10.1016/S0960-9822(98)70088-0
39. Yamamoto A. Pds1p is required for faithful execution of anaphase in the yeast, Saccharomyces cerevisiae. J Cell Biol. 1996;133: 85–97. doi:10.1083/jcb.133.1.85
40. Shirayama M, Matsui Y, Toh-E A. The Yeast *TEM1* Gene, Which Encodes a GTP-Binding Protein, Is Involved in Termination of M Phase. Mol Cell Biol. 1994;14: 7476–7482. doi:10.1128/mcb.14.11.7476-7482.1994
41. Visintin R, Hwang ES, Amon A. Cfi1 prevents premature exit from mitosis by anchoring Cdc14 phosphatase in the nucleolus. Nature. 1999;398: 818–823. doi:10.1038/19775
42. Visintin R, Craig K, Hwang ES, Prinz S, Tyers M, Amon A. The Phosphatase Cdc14 Triggers Mitotic Exit by Reversal of Cdk-Dependent Phosphorylation. Mol Cell. 1998;2: 709–718. doi:10.1016/S1097-2765(00)80286-5
43. Wang Y, Burke DJ. Cdc55p, the B-type regulatory subunit of protein phosphatase 2A, has multiple functions in mitosis and is required for the kinetochore/spindle checkpoint in Saccharomyces cerevisiae. Mol Cell Biol. 1997;17: 620–626. doi:10.1128/MCB.17.2.620
44. Shirayama M, Zachariae W, Ciosk R, Nasmyth K. The Polo-like kinase Cdc5p and the WD-repeat protein Cdc20p/fizzy are regulators and substrates of the anaphase promoting complex in Saccharomyces cerevisiae. EMBO J. 1998;17: 1336–1349. doi:10.1093/emboj/17.5.1336
45. Uhlmann F, Lottspeich F, Nasmyth K. Sister-chromatid separation at anaphase onset is promoted by cleavage of the cohesin subunit Scc1. Nature. 1999;400: 37–42. doi:10.1038/21831
46. Rudner AD, Murray AW. Phosphorylation by Cdc28 Activates the Cdc20-Dependent Activity of the Anaphase-Promoting Complex. J Cell Biol. 2000;149: 1377–1390. doi:10.1083/jcb.149.7.1377
