# Supplementary material for: Modeling the START transition in the budding yeast cell cycle
Source: PLoS Comput Biol. 2024 Aug 2;20(8):e1012048. doi: 10.1371/journal.pcbi.1012048 (PMC11324117; doi:10.1371/journal.pcbi.1012048)
Supplement: S1 Text — (PDF) [file pcbi.1012048.s016.pdf]

S1 Text. List of abbreviations

DNA – Deoxy-Ribo Nucleic Acid
S, M, G1, G2 – Phases of cell cycle: DNA Synthesis, Mitosis, Gap phases 1, 2
CDK – Cyclin-Dependent Kinase
CKI – Cyclin-dependent Kinase Inhibitor
SBF, MBF – Scb- and Mcb-element Binding Factor
R point – Restriction Point
BYCC – Chen et al, 2004, Budding Yeast Cell Cycle model
START-BYCC – New START model incorporated with BYCC
ORI – species in our model, which is a marker for Origin of replication
WT – Wild Type
BUD – species in our model, which is a marker for Budding
SBFB – SBF Bound to promoter
WSB – Whi5-SBF Bound to promoter
Swi4B – Swi4 dimer Bound to promoter
SBFa1-a5 – explained in legend of Figure 4E.
