# Supplementary material for: Modeling the START transition in the budding yeast cell cycle
Source: PLoS Comput Biol. 2024 Aug 2;20(8):e1012048. doi: 10.1371/journal.pcbi.1012048 (PMC11324117; doi:10.1371/journal.pcbi.1012048)
Supplement: S2 Text — (PDF) [file pcbi.1012048.s017.pdf]

S2 Text. Equations, parameters, and initial conditions

Also available here: https://github.com/jravilab/start-bycc

START BYCC Model Differential Equations

Functions
$Mass_{Act}(k_1) = k_1$
$Mass_{0001}(k_1, S_1) = k_1 \times S_1$
$Mass_{0002}(k_1, S_1, S_2) = k1 \times S_1 \times S_2$
$Michaeli(M_1, J_1, k_1, S_1) = \frac{k_1 \times M_1 \times S_1}{(J_1 + S_1)}$
$BB(A_1, A_2, A_3, A_4) = A_2 - A_1 + (A_3 \times A_2) + (A_4 \times A_1)$
$GK(A_1, A_2, A_3, A_4) = \frac{2.0 \times A_4 \times A_1}{BB(A_1,A_2,A_3,A_4) + \sqrt{(BB(A_1,A_2,A_3,A_4)^2 - 4.0 \times ((A_2 - A_1) \times A_4 \times A_1))}}$

Compartment Equations

Variables
$T19 = 1.0 \times IEP + 1.0 \times IE$
$T18 = 1.0 \times TEM1GDP + 1.0 \times TEM1GTP$
$T17 = 1.0 \times CDC15 + 1.0 \times CDC15i$
$T16 = 1.0 \times ESP1 + 1.0 \times PE$
$T15 = 1.0 \times PROM2 + 1.0 \times SBFB6PQ + 1.0 \times SBFB6P + 1.0 \times SBFB + 1.0 \times SWI4B + 1.0 \times W4B + 1.0 \times WSB56P + 1.0 \times WSB5P + 1.0 \times WSB6PQ + 1.0 \times WSB6P + 1.0 \times WSB$
$T14 = 1.0 \times SBFB6PQ + 1.0 \times SBFB6P + 1.0 \times SBFB + 1.0 \times SBFF46PQ + 1.0 \times SBFF46P + 1.0 \times SBFF4P + 1.0 \times SBFF6PQ + 1.0 \times SBFF6P + 1.0 \times SBFF + 1.0 \times SWI4B + 4.0 \times SWI4C + 1.0 \times SWI4F + 4.0 \times SWI4PC + 1.0 \times SWI4P + 1.0 \times SWI4 + 1.0 \times W4B + 1.0 \times WSB56P + 1.0 \times WSB5P + 1.0 \times WSB6PQ + 1.0 \times WSB6P + 1.0 \times WSB + 1.0 \times WSF45P + 1.0 \times WSF46PQ + 1.0 \times WSF46P + 1.0 \times WSF4P + 1.0 \times WSF56P + 1.0 \times WSF5P + 1.0 \times WSF6PQ + 1.0 \times WSF6P + 1.0 \times WSF$
$T13 = 1.0 \times W4B + 4.0 \times WHI5C + 4.0 \times WHI5PC + 1.0 \times WHI5PN + 1.0 \times WHI5 + 1.0 \times WMB + 1.0 \times WSB56P + 1.0 \times WSB5P + 1.0 \times WSB6PQ + 1.0 \times WSB6P + 1.0 \times WSB + 1.0 \times WSF45P + 1.0 \times WSF46PQ + 1.0 \times WSF46P + 1.0 \times WSF4P + 1.0 \times WSF56P + 1.0 \times WSF5P + 1.0 \times WSF6PQ + 1.0 \times WSF6P + 1.0 \times WSF$
$T12 = 1.0 \times MBFa + 1.0 \times MBFi + 1.0 \times MBFo + 1.0 \times MBFp + 1.0 \times MBFpo + 1.0 \times PROM5 + 1.0 \times WMB$
$T11 = 1.0 \times MBFF + 1.0 \times MBFa + 1.0 \times MBFi + 1.0 \times MBFo + 1.0 \times MBFp + 1.0 \times MBFpo + 1.0 \times MBP1 + 1.0 \times WMB$

Variables
$T10 = 1.0 \times MBFF + 1.0 \times MBFa + 1.0 \times MBFi + 1.0 \times MBFo + 1.0 \times MBFp + 1.0 \times MBFpo + 1.0 \times SBFB6PQ + 1.0 \times SBFB6P + 1.0 \times SBFB + 1.0 \times SBFF46PQ + 1.0 \times SBFF46P + 1.0 \times SBFF4P + 1.0 \times SBFF6PQ + 1.0 \times SBFF6P + 1.0 \times SBFF + 4.0 \times SWI6C + 4.0 \times SWI6PQC + 1.0 \times SWI6PQ + 1.0 \times SWI6P + 4.0 \times SWI6QC + 1.0 \times SWI6 + 1.0 \times WMB + 1.0 \times WSB56P + 1.0 \times WSB5P + 1.0 \times WSB6PQ + 1.0 \times WSB6P + 1.0 \times WSB + 1.0 \times WSF45P + 1.0 \times WSF46PQ + 1.0 \times WSF46P + 1.0 \times WSF4P + 1.0 \times WSF56P + 1.0 \times WSF5P + 1.0 \times WSF6PQ + 1.0 \times WSF6P + 1.0 \times WSF$
$T9 = 1.0 \times IEP + 1.0 \times IE$
$T8 = 1.0 \times TEM1GDP + 1.0 \times TEM1GTP$
$T7 = 1.0 \times CDC15 + 1.0 \times CDC15i$
$T6 = 1.0 \times ESP1 + 1.0 \times PE$
$T5 = 1.0 \times PROM2 + 1.0 \times SBFB6PQ + 1.0 \times SBFB6P + 1.0 \times SBFB + 1.0 \times SWI4B + 1.0 \times W4B + 1.0 \times WSB56P + 1.0 \times WSB5P + 1.0 \times WSB6PQ + 1.0 \times WSB6P + 1.0 \times WSB$
$T4 = 1.0 \times SBFB6PQ + 1.0 \times SBFB6P + 1.0 \times SBFB + 1.0 \times SBFF46PQ + 1.0 \times SBFF46P + 1.0 \times SBFF4P + 1.0 \times SBFF6PQ + 1.0 \times SBFF6P + 1.0 \times SBFF + 1.0 \times SWI4B + 4.0 \times SWI4C + 1.0 \times SWI4F + 4.0 \times SWI4PC + 1.0 \times SWI4P + 1.0 \times SWI4 + 1.0 \times W4B + 1.0 \times WSB56P + 1.0 \times WSB5P + 1.0 \times WSB6PQ + 1.0 \times WSB6P + 1.0 \times WSB + 1.0 \times WSF45P + 1.0 \times WSF46PQ + 1.0 \times WSF46P + 1.0 \times WSF4P + 1.0 \times WSF56P + 1.0 \times WSF5P + 1.0 \times WSF6PQ + 1.0 \times WSF6P + 1.0 \times WSF$
$T3 = 1.0 \times W4B + 4.0 \times WHI5C + 4.0 \times WHI5PC + 1.0 \times WHI5PN + 1.0 \times WHI5 + 1.0 \times WMB + 1.0 \times WSB56P + 1.0 \times WSB5P + 1.0 \times WSB6PQ + 1.0 \times WSB6P + 1.0 \times WSB + 1.0 \times WSF45P + 1.0 \times WSF46PQ + 1.0 \times WSF46P + 1.0 \times WSF4P + 1.0 \times WSF56P + 1.0 \times WSF5P + 1.0 \times WSF6PQ + 1.0 \times WSF6P + 1.0 \times WSF$
$T2 = 1.0 \times MBFa + 1.0 \times MBFi + 1.0 \times MBFo + 1.0 \times MBFp + 1.0 \times MBFpo + 1.0 \times PROM5 + 1.0 \times WMB$
$T1 = 1.0 \times MBFF + 1.0 \times MBFa + 1.0 \times MBFi + 1.0 \times MBFo + 1.0 \times MBFp + 1.0 \times MBFpo + 1.0 \times MBP1 + 1.0 \times WMB$
$T0 = 1.0 \times MBFF + 1.0 \times MBFa + 1.0 \times MBFi + 1.0 \times MBFo + 1.0 \times MBFp + 1.0 \times MBFpo + 1.0 \times SBFB6PQ + 1.0 \times SBFB6P + 1.0 \times SBFB + 1.0 \times SBFF46PQ + 1.0 \times SBFF46P + 1.0 \times SBFF4P + 1.0 \times SBFF6PQ + 1.0 \times SBFF6P + 1.0 \times SBFF + 4.0 \times SWI6C + 4.0 \times SWI6PQC + 1.0 \times SWI6PQ + 1.0 \times SWI6P + 4.0 \times SWI6QC + 1.0 \times SWI6 + 1.0 \times WMB + 1.0 \times WSB56P + 1.0 \times WSB5P + 1.0 \times WSB6PQ + 1.0 \times WSB6P + 1.0 \times WSB + 1.0 \times WSF45P + 1.0 \times WSF46PQ + 1.0 \times WSF46P + 1.0 \times WSF4P + 1.0 \times WSF56P + 1.0 \times WSF5P + 1.0 \times WSF6PQ + 1.0 \times WSF6P + 1.0 \times WSF$
$IET = 1.0 \times IEP + 1.0 \times IE$
$TEM1T = 1.0 \times TEM1GDP + 1.0 \times TEM1GTP$
$CDC15T = 1.0 \times CDC15 + 1.0 \times CDC15i$
$ESP1T = 1.0 \times ESP1 + 1.0 \times PE$
$PROM2T = 1.0 \times PROM2 + 1.0 \times SBFB6PQ + 1.0 \times SBFB6P + 1.0 \times SBFB + 1.0 \times SWI4B + 1.0 \times W4B + 1.0 \times WSB56P + 1.0 \times WSB5P + 1.0 \times WSB6PQ + 1.0 \times WSB6P + 1.0 \times WSB$

Variables
$SWI4T = 1.0 \times SBFB6PQ + 1.0 \times SBFB6P + 1.0 \times SBFB + 1.0 \times SBFF46PQ + 1.0 \times SBFF46P + 1.0 \times SBFF4P + 1.0 \times SBFF6PQ + 1.0 \times SBFF6P + 1.0 \times SBFF + 1.0 \times SWI4B + 4.0 \times SWI4C + 1.0 \times SWI4F + 4.0 \times SWI4PC + 1.0 \times SWI4P + 1.0 \times SWI4 + 1.0 \times W4B + 1.0 \times WSB56P + 1.0 \times WSB5P + 1.0 \times WSB6PQ + 1.0 \times WSB6P + 1.0 \times WSB + 1.0 \times WSF45P + 1.0 \times WSF46PQ + 1.0 \times WSF46P + 1.0 \times WSF4P + 1.0 \times WSF56P + 1.0 \times WSF5P + 1.0 \times WSF6PQ + 1.0 \times WSF6P + 1.0 \times WSF$
$WHI5T = 1.0 \times W4B + 4.0 \times WHI5C + 4.0 \times WHI5PC + 1.0 \times WHI5PN + 1.0 \times WHI5 + 1.0 \times WMB + 1.0 \times WSB56P + 1.0 \times WSB5P + 1.0 \times WSB6PQ + 1.0 \times WSB6P + 1.0 \times WSB + 1.0 \times WSF45P + 1.0 \times WSF46PQ + 1.0 \times WSF46P + 1.0 \times WSF4P + 1.0 \times WSF56P + 1.0 \times WSF5P + 1.0 \times WSF6PQ + 1.0 \times WSF6P + 1.0 \times WSF$
$PROM5T = 1.0 \times MBFa + 1.0 \times MBFi + 1.0 \times MBFo + 1.0 \times MBFp + 1.0 \times MBFpo + 1.0 \times PROM5 + 1.0 \times WMB$
$MBP1T = 1.0 \times MBFF + 1.0 \times MBFa + 1.0 \times MBFi + 1.0 \times MBFo + 1.0 \times MBFp + 1.0 \times MBFpo + 1.0 \times MBP1 + 1.0 \times WMB$
$SWI6T = 1.0 \times MBFF + 1.0 \times MBFa + 1.0 \times MBFi + 1.0 \times MBFo + 1.0 \times MBFp + 1.0 \times MBFpo + 1.0 \times SBFB6PQ + 1.0 \times SBFB6P + 1.0 \times SBFB + 1.0 \times SBFF46PQ + 1.0 \times SBFF46P + 1.0 \times SBFF4P + 1.0 \times SBFF6PQ + 1.0 \times SBFF6P + 1.0 \times SBFF + 4.0 \times SWI6C + 4.0 \times SWI6PQC + 1.0 \times SWI6PQ + 1.0 \times SWI6P + 4.0 \times SWI6QC + 1.0 \times SWI6 + 1.0 \times WMB + 1.0 \times WSB56P + 1.0 \times WSB5P + 1.0 \times WSB6PQ + 1.0 \times WSB6P + 1.0 \times WSB + 1.0 \times WSF45P + 1.0 \times WSF46PQ + 1.0 \times WSF46P + 1.0 \times WSF4P + 1.0 \times WSF56P + 1.0 \times WSF5P + 1.0 \times WSF6PQ + 1.0 \times WSF6P + 1.0 \times WSF$
$D = \frac{1.026}{\mu} - 32.0$
$F = e^{(-\mu) \times D}$
$YDJ1 = k_{ydj1} \times MASS$
$SSA1 = (k_{ssa0} + k_{ssab2} \times CLB2 + k_{ssaw5} \times SWI5) \times \frac{90.0}{mdt})$
$V_{acln3} = k_{gkcln3} \times YDJ1$
$MCM1 = GK(k_{amcm} \times CLB2, k_{imcm}, J_{amcm}, J_{imcm})$
$V_{db2} = k_{db2_p} + k_{db2_{pp}} \times CDH1 + kdb2_{ppp} \times CDC20$
$V_{db5} = k_{db5_p} + k_{db5_{pp}} \times CDC20$
$V_{d2c1} = k_{d2c1} \times (ec1n3 \times CLN3 + ec1k2 \times BCK2 + ec1n2 \times CLN2 + ec1b5 \times CLB5 + ec1b2 \times CLB2)$
$V_{d2f6} = k_{d2f6} \times (ef6n3 \times CLN3 + ef6k2 \times BCK2 + ef6n2 \times CLN2 + ef6b5 \times CLB5 + ef6b2 \times CLB2)$
$V_{ppc1} = k_{ppc1} \times CDC14$
$V_{ppf6} = k_{ppf6} \times CDC14$
$V_{kpc1} = \frac{k_{d1c1} + V_{d2c1}}{(J_{d2c1} + SIC1 + C2 + C5)}$

Variables (cont.)
$V_{aiep} = k_{aiep} \times CLB2$
$V_{d20} = k_{d20} + k_{d20_p} \times CDH1$
$V_{acdh} = k_{acdh_p} + k_{acdh_{pp}} \times CDC14$
$Vicdh = k_{icdh_p} + k_{icdh_{pp}} \times (eicdhn3 \times CLN3 + eicdhn2 \times CLN2 + eicdhb5 \times CLB5 + eicdhb2 \times CLB2)$
$V_{kpnet} = k_{pnet_p} + k_{pnet_{pp}} \times CDC15$
$PP2A = \frac{PP2AT \times (1.0 + k_i \times k_{pp} \times ESP1)}{(1.0 + k_i \times ESP1)}$
$V_{ppnet} = k_{ppnet_p} + k_{ppn_{0001}} \times PP2A$
$V_{dpds} = k_{dpds_p} + k_{dpds_{pp}} \times CDC20 + k_{dpd_{0001}} \times CDH1$
$Vpn = epn3 \times CLN3 + epn2 \times CLN2 + epb5 \times CLB5 + epk2 \times BCK2$
$V_{pcln} = V_{pnmax} \times \frac{V_{pn}^N}{(J_{pn}^N + V_{pn}^N)}$
$V_{ppcln} = k_{ppcln} + k_{ppcln_p} \times CDC14$
$V_{pnm} = epn3m \times CLN3 + epn2m \times CLN2 + epb5m \times CLB5 + epk2m \times BCK2$
$V_{pclnm} = \frac{V_{pmaxm} \times V_{pnm}^N}{(J_{pn}^N + V_{pnm}^N))}$
$V_{pnw} = epn3w \times CLN3 + epn2w \times CLN2 + epb5w \times CLB5 + epk2w \times BCK2$
$V_{pclnw} = \frac{V_{nmaxw} \times V_{pnw}^N}{(J_{pn}^N + V_{pnw}^N))}$
$V_{pclb} = k_{p_p} + k_{p_{pp}} \times CLB2$
$V_{pp14} = k_{pp14} \times CDC14$
$V_{pclb26} = k_{p_p} + k_{p_pp} \times CLB2 + epb5q \times CLB5$
$V_{ppase} = PPase$
$CLB2T = CLB2 + C2 + F2 + C2P + F2P$
$CLB5T = CLB5 + C5 + F5 + C5P + F5P$
$CDC14T = CDC14 + RENT + RENTP$
$NET1T = NET1 + NET1P + RENT + RENTP$
$SIC1T = SIC1 + C2 + C5 + SIC1P + C2P + C5P$
$CDC6T = CDC6 + F2 + F5 + CDC6P + F2P + F5P$

Variables (cont.)
$KIT = SIC1T + CDC6T$
$SBFact = k_{asbf1} \times SBFB + k_{asbf2} \times (SBFB6P + SBFB6PQ) + k_{asbf3} \times (WSB6P + WSB6PQ) + k_{asbf4} \times WSB5P + k_{asbf5} \times SWI4B$
$MBFact = MBFa$
$SWI6CTOT = SWI6C + SWI6QC + SWI6PQC$
$SBFa1 = k_{asbf1} \times SBFB$
$SBFa2 = k_{asbf2} \times (SBFB6P + SBFB6PQ)$
$SBFa3 = k_{asbf3} \times (WSB6P + WSB6PQ)$
$SBFa4 = k_{asbf4} \times WSB5P$
$SBFa5 = k_{asbf5} \times SWI4B$
$SWI4cycf = 1.0 - SWI4nucf$
$WHI5cycf = 1.0 - WHI5nucf$
$SWI6cycf = 1.0 - SWI6nucf$
$CLN310x = 10.0 \times CLN3$

Conditional variables
$CLN3 = \begin{cases} 0.0 & ,if \quad CLN3T = 0.0 \\ CLN3T \times MASS \times GK(Vacln3, SSA1, Jacln3 \times CLN3T, Jicln3 \times CLN3T) & ,otherwise \end{cases}$
$BCK2 = \begin{cases} 0.0 & ,if \quad BCK2T = 0.0 \\ BCK2T \times MASS \times GK(Va_{cln3}, SSA1, J_{abck2} \times BCK2T, J_{ibck2} \times BCK2T & ,otherwise \end{cases}$
$SWI4nucf = \begin{cases} 0.0 & ,if \quad SWI4T < 1.0 \times 10^{-8} \\ \frac{SWI4T - 4.0 \times (SWI4C + SWI4PC))}{SWI4T} & ,otherwise \end{cases}$
$WHI5nucf = \begin{cases} 0.0 & ,if \quad WHI5T < 1.0 \times 10^{-8} \\ \frac{WHI5T - 4.0 \times (WHI5C + WHI5PC))}{WHI5T} & ,otherwise \end{cases}$
$SWI6nucf = \begin{cases} 0.0 & ,if \quad SWI6T < 1.0 \times 10^{-8} \\ \frac{SWI6T - 4.0 \times (SWI6C + SWI6QC + SWI6PQC))}{SWI6T} & ,otherwise \end{cases}$

Parameter Equations
$\mu = \frac{log(2.0)}{mdt}$

Independent Species

Differential Equations
$\frac{d}{dt}MASS = (\mu \times MASS \times (1.0 - \frac{MASS}{MASS_{max}})$
$\frac{d}{dt}CLN2 = (k_{sn2_p} + k_{sn2_{pp}} \times SBFact + ksn2_{ppp} \times MBFact) - (kdn2 \times CLN2)$
$\frac{d}{dt}CLB5 = ((k_{sb5_p} + k_{sb5_{pp}} \times SBFact + k_{sb5_{ppp}} \times MBFact) \times MASS) - (V_{db5} \times CLB5) - (k_{asb5} \times CLB5 \times SIC1) + (k_{dib5} \times C5) + (k_{d3c1} \times C5P) - (k_{asf5} \times CLB5 \times CDC6) + (k_{dif5} \times F5) + (k_{d3f6} \times F5P)$
$\frac{d}{dt}CLB2 = ((k_{sb2_p} + k_{sb2_{pp}} \times MCM1) \times MASS) - (V_{db2} \times CLB2) - (k_{asb2} \times CLB2 \times SIC1) + (k_{dib2} \times C2) + (k_{d3c1} \times C2P) - (k_{asf2} \times CLB2 \times CDC6) + (k_{dif2} \times F2) + (k_{d3f6} \times F2P)$
$\frac{d}{dt}SIC1 = (k_{sc1_p} + k_{sc1_{pp}} \times SWI5) - (k_{dc1} \times SIC1) - (V_{kpc1} \times SIC1) + (V_{ppc1} \times SIC1P) - (k_{asb2} \times CLB2 \times SIC1) + (k_{dib2} \times C2) - (k_{asb5} \times CLB5 \times SIC1) + (k_{dib5} \times C5) + (V_{db2} \times C2) + (V_{db5} \times C5)$
$\frac{d}{dt}SIC1P = (V_{kpc1} \times SIC1) - (V_{ppc1} \times SIC1P) - (k - d3c1 \times SIC1P) + (V_{db2} \times C2P) + (V_{db5} \times C5P)$
$\frac{d}{dt}C2 = (k_{asb2} \times CLB2 \times SIC1) - (k_{dib2} \times C2) - (V_{kpc1} \times C2) + (V_{ppc1} \times C2P) - (V_{db2} \times C2)$
$\frac{d}{dt}C5 = (k_{asb5} \times CLB5 \times SIC1) - (k_{dib5} \times C5) - (V_{kpc1} \times C5) + (V_{ppc1} \times C5P) - (V_{db5} \times C5)$
$\frac{d}{dt}C2P = (V_{kpc1} \times C2) - (V_{ppc1} \times C2P) - (k_{d3c1} \times C2P) - (V_{db2} \times C2P)$
$\frac{d}{dt}C5P = (V_{kpc1} \times C5) - (V_{ppc1} \times C5P) - (k_{d3c1} \times C5P) - (Vdb5 \times C5P)$
$\frac{d}{dt}CDC6 = (k_{sf6_p} + k_{sf6_{pp}} \times SWI5 + k_{sf6_{ppp}} \times SBFact) - (k_{df6} \times CDC6) - (V_{kpf6} \times CDC6) + (V_{ppf6} \times CDC6P) - (k_{asf2} \times CLB2 \times CDC6) + (k_{dif2} \times F2) - (k_{asf5} \times CLB5 \times CDC6) + (k_{dif5} \times F5) + (V_{db2} \times F2) + (V_{db5} \times F5)$
$\frac{d}{dt}CDC6P = (V_{kpf6} \times CDC6) - (V_{ppf6} \times CDC6P) - (k_{d3f6} \times CDC6P) + (V_{db2} \times F2P) + (V_{db5} \times F5P)$
$\frac{d}{dt}F2 = (k_{asf2} \times CLB2 \times CDC6) - (k_{dif2} \times F2) - (V_{kpf6} \times F2) + (V_{ppf6} \times F2P) - (V_{db2} \times F2)$
$\frac{d}{dt}F5 = (k_{asf5} \times CLB5 \times CDC6) - (k_{dif5} \times F5) - (Vkpf6 \times F5) + (Vppf6 \times F5P) - (Vdb5 \times F5)$
$\frac{d}{dt}F2P = (V_{kpf6} \times F2) - (V_{ppf6} \times F2P) - (k_{d3f6} \times F2P) - (V_{db2} \times F2P)$
$\frac{d}{dt}F5P = (V_{kpf6} \times F5) - (V_{ppf6} \times F5P) - (k_{d3f6} \times F5P) - (V_{db5} \times F5P)$
$SWI5 = (k_{sswi_p} + k_{sswi_{pp}} \times MCM1) - (k_{dswi} \times SWI5) - ((k_{iswi} \times CLB2) \times SWI5) + (k_{aswi} \times CDC14) \times SWI5P$
$\frac{d}{dt}SWI5P = (k_{iswi} \times CLB2) \times SWI5 - ((k_{aswi} \times CDC14) \times SWI5P) - (k_{dswi} \times SWI5P)$
$\frac{d}{dt}IE = -(V_{aiep} \times IE \times 1.0/(J_{aiep} + IE)) + (k_{iiep} \times IEP \times 1.0/(J_{iiep} + IEP))$
$\frac{d}{dt}IEP = \frac{V_{aiep} \times IE}{J_{aiep}+IE} - \frac{k_{iiep} \times IEP}{J_{iiep}+IEP}$
$\frac{d}{dt}CDC20i = (k_{s20_p} + k_{s20_{pp}} \times MCM1) - (V_{d20} \times CDC20i) - ((k_{a20_p} + k_{a20_{pp}} \times IEP) \times CDC20i) + (MAD2 \times CDC20)$
$\frac{d}{dt}CDC20 = (k_{a20_p} + k_{a20_{pp}} \times IEP) \times CDC20i - (MAD2 \times CDC20) - (V_{d20} \times CDC20)$

Differential Equations (cont.)

$\frac{d}{dt}CDH1 = k_{scdh} - (k_{dcdh} \times CDH1) - \frac{V_{icdh} \times CDH1}{J_{icdh}+CDH1} + \frac{V_{acdh} \times CDH1i}{J_{acdh}+CDH1i}$

$\frac{d}{dt}CDH1i = \frac{V_{icdh} \times CDH1}{J_{icdh}+CDH1} - (Vacdh \times CDH1i \times 1.0/(Jacdh + CDH1i)) - (kdcdh \times CDH1i)$

$\frac{d}{dt}CDC14 = k_{s14} - (k_{d14} \times CDC14) - (k_{asrent} \times CDC14 \times NET1) + (k_{dirent} \times RENT) - (k_{asrentp} \times CDC14 \times NET1P) + (k_{direntp} \times RENTP) + (k_{dnet} \times RENT) + (k_{dnet} \times RENTP)$

$\frac{d}{dt}NET1 = (k_{snet}) - (k_{dnet} \times NET1) - (V_{kpnet} \times NET1) + (V_{ppnet} \times NET1P) - (k_{asrent} \times CDC14 \times NET1) + (k_{dirent} \times RENT) + (k_{d14} \times RENT)$

$\frac{d}{dt}RENT = (k_{asrent} \times CDC14 \times NET1) - (k_{dirent} \times RENT) - (V_{kpnet} \times RENT) + (V_{ppnet} \times RENTP) - (k_{dnet} \times RENT) - (k_{d14} \times RENT)$

$\frac{d}{dt}NET1P = (V_{kpnet} \times NET1) - (V_{ppnet} \times NET1P) - (k_{dnet} \times NET1P) - (k_{asrent_p} \times CDC14 \times NET1P) + (k_{dirent_p} \times RENTP) + (k_{d14} \times RENTP)$

$\frac{d}{dt}RENTP = (k_{asrent_p} \times CDC14 \times NET1P) - (k_{dirent_p} \times RENTP) + (V_{kpnet} \times RENT) - (V_{ppnet} \times RENTP) - (k_{dnet} \times RENTP) - (k_{d14} \times RENTP)$

$\frac{d}{dt}TEM1GDP = \frac{-LTE1 \times TEM1GDP}{J_{atem}+TEM1GDP} + \frac{BUB2 \times TEM1GTP}{J_{item}+TEM1GTP}$

$\frac{d}{dt}TEM1GTP = \frac{LTE1 \times TEM1GDP}{J_{atem}+TEM1GDP} - \frac{BUB2 \times TEM1GTP}{J_{item}+TEM1GTP}$

$\frac{d}{dt}CDC15i = -k_{a15_p} \times TEM1GDP + k_{a15_{pp}} \times TEM1GTP + k_{a15_{ppp}} \times CDC14 \times CDC15i + k_{i15} \times CDC15$

$\frac{d}{dt}CDC15 = (k_{a15_p} \times TEM1GDP) + (k_{a15_{pp}} \times TEM1GTP) + (k_{a15_{ppp}} \times CDC14 \times CDC15i) - (k_{i15} \times CDC15)$

$\frac{d}{dt}PDS1 = (k_{spds_p}) - (V_{dpds} \times PDS1) - (k_{asesp} \times PDS1 \times ESP1) + (k_{diesp} \times PE)$

$\frac{d}{dt}ESP1 = -(k_{asesp} \times PDS1 \times ESP1) + (k_{diesp} \times PE) + (V_{dpds} \times PE)$

$\frac{d}{dt}PE = (k_{asesp} \times PDS1 \times ESP1) - (k_{diesp} \times PE) - (V_{dpds} \times PE)$

$\frac{d}{dt}ORI = (k_{sori} \times (eorib5 \times CLB5 + eorib2 \times CLB2)) - (k_{dori} \times ORI)$

$\frac{d}{dt}BUD = (k_{sbud} \times (ebudn2 \times CLN2 + ebudn3 \times CLN3 + ebudb5 \times CLB5)) - (k_{dbud} \times BUD)$

$\frac{d}{dt}SPN = \frac{k_{sspn} \times CLB2}{J_{spn}+CLB2-(k_{dspn} \times SPN)}$

$\frac{d}{dt}MAD2 = 0.0$

$\frac{d}{dt}LTE1 = 0.0$

$\frac{d}{dt}BUB2 = 0.0$

$\frac{d}{dt}SWI4 = -(k_{as46} \times SWI4 \times SWI6) + (k_{di46} \times SBFF) - ((k_{sbs4} \times BCK2) \times SWI4) + (k_{dbs4} \times SWI4F) - (k_{as46} \times SWI4 \times SWI6P) + (k_{di46} \times SBFF6P) - (k_{as46} \times SWI4 \times SWI6PQ) + (k_{di46} \times SBFF6PQ) - ((ef4p \times V_{pclb}) \times SWI4) + (V_{pp14} \times SWI4P) + (k_{imp} \times SWI4C) \times \frac{cytoplas}{nucleus}$

Differential Equations (cont.)

$\frac{d}{dt}SWI6 =$
$-(k_{as46} \times SWI4 \times SWI6) + (k_{di46} \times SBFF) - ((ef6p \times V_{pcln}) \times SWI6) + (V_{ppcln} \times SWI6P) - (k_{as46} \times SWI4P \times SWI6) + (k_{di46} \times SBFF4P) + (k_{imp} \times SWI6C) \times \frac{cytoplas}{nucleus} - (k_{asmbf} \times MBP1 \times SWI6) + (k_{dimbf} \times MBFF)$

$\frac{d}{dt}WHI5 = -(k_{asws} \times WHI5 \times SBFF) + (k_{diws} \times WSF) - (k_{asws} \times WHI5 \times SBFB) + (k_{diws} \times WSB) - (k_{asw4} \times SWI4B \times WHI5) + (k_{diw4} \times W4B) - ((ef5p \times V_{pclnw}) \times WHI5) + (V_{ppcln} \times WHI5PN) - (k_{asws} \times WHI5 \times SBFF6P) + (k_{diws} \times WSF6P) - (k_{asws} \times WHI5 \times SBFF6PQ) + (k_{diws} \times WSF6PQ) + (k_{imp} \times WHI5C) \times \frac{cytoplas}{nucleus} - (k_{aswm} \times MBFa \times WHI5) + (k_{diwm} \times WMB)$

$\frac{d}{dt}MBP1 = -(k_{asmbf} \times MBP1 \times SWI6) + (k_{dimbf} \times MBFF)$

$\frac{d}{dt}PROM2 = -(k_{asprom} \times SBFF \times PROM2) + (k_{diprom} \times SBFB) - (k_{asprom} \times WSF \times PROM2) + (k_{diprom} \times WSB) - (k_{asprom} \times SWI4F \times PROM2) + (k_{diprom} \times SWI4B) + (ef4p \times V_{pclb}) \times SBFB + (ef4p \times V_{pclb}) \times SBFB6P + (ef4p \times V_{pclb}) \times SBFB6PQ + (ef4p \times V_{pclb}) \times WSB + (ef4p \times V_{pclb}) \times WSB5P + (ef4p \times V_{pclb}) \times WSB6P + (ef4p \times V_{pclb}) \times WSB6PQ + (V_{pclb} \times SWI4B)$

$\frac{d}{dt}PROM5 = -(k_{asprom} \times MBFF \times PROM5) + (k_{diprom} \times MBFi)$

$\frac{d}{dt}NRM1/dt = (k_{snrm1_p} \times MBFact) - (kdnrm1 \times NRM1)$

$\frac{d}{dt}SWI4C =$
$(MSN5 \times SBFF6PQ) \times \frac{nucleus}{cytoplas} + (MSN5 \times WSF6PQ) \times \frac{nucleus}{cytoplas} + (V_{ppase} \times SWI4PC) - (k_{imp} \times SWI4C)$

$\frac{d}{dt}SWI4P = (MSN5 \times SBFF46PQ) \times \frac{nucleus}{cytoplas} + (MSN5 \times WSF46PQ) \times \frac{nucleus}{cytoplas} + (MSN5 \times WSF45P) \times \frac{nucleus}{cytoplas} - (V_{ppase} \times SWI4PC)$

$\frac{d}{dt}SWI4P =$
$-(k_{as46} \times SWI4P \times SWI6) + (k_{di46} \times SBFF4P) - (k_{as46} \times SWI4P \times SWI6P) + (k_{di46} \times SBFF46P) - (k_{as46} \times SWI4P \times SWI6PQ) + (k_{di46} \times SBFF46PQ) + (ef4p \times V_{pclb}) \times SWI4 - (V_{pp14} \times SWI4P) + (V_{pclb} \times SWI4B)$

$\frac{d}{dt}SWI4B = (k_{asprom} \times SWI4F \times PROM2) - (k_{diprom} \times SWI4B) - (k_{asw4} \times SWI4B \times WHI5) + (k_{diw4} \times W4B) + (ef5p \times V_{pclnw}) \times W4B - (V_{pclb} \times SWI4B)$

$\frac{d}{dt}SWI4F = (k_{sbs4} \times BCK2) \times SWI4 - (k_{dbs4} \times SWI4F) - (k_{asprom} \times SWI4F \times PROM2) + (k_{diprom} \times SWI4B)$

$\frac{d}{dt}W4B = (k_{asw4} \times SWI4B \times WHI5) - (k_{diw4} \times W4B) - ((ef5p \times V_{pclnw}) \times W4B)$

$\frac{d}{dt}SBFF = (k_{as46} \times SWI4 \times SWI6) - (k_{di46} \times SBFF) - (k_{asws} \times WHI5 \times SBFF) + (k_{diws} \times WSF) - (k_{asprom} \times SBFF \times PROM2) + (k_{diprom} \times SBFB) - ((ef6p \times V_{pcln}) \times SBFF) + (V_{ppcln} \times SBFF6P) + (k_{diwp} \times WSF5P) - ((ef4p \times V_{pclb}) \times SBFF) + (V_{pp14} \times SBFF4P)$

$\frac{d}{dt}SBFF4P = (k_{as46} \times SWI4P \times SWI6) - (k_{di46} \times SBFF4P) + (ef4p \times V_{pclb}) \times SBFF - (V_{pp14} \times SBFF4P) + (V_{ppcln} \times SBFF46P) + (ef4p \times V_{pclb}) \times SBFB - ((ef6p \times V_{pcln}) \times SBFF4P)$

$\frac{d}{dt}SBFF6P = (ef6p \times V_{pcln}) \times SBFF - (V_{ppcln} \times SBFF6P) + (k_{diwp} \times WSF56P) - ((ef6q \times V_{pclb26}) \times SBFF6P) + (V_{pp14} \times SBFF6PQ) + (k_{as46} \times SWI4 \times SWI6P) - (k_{di46} \times SBFF6P) - (k_{asws} \times WHI5 \times SBFF6P) + (k_{diws} \times WSF6P) - ((ef4p \times V_{pclb}) \times SBFF6P) + (V_{pp14} \times SBFF46P)$

$\frac{d}{dt}SBFF6PQ = (ef6q \times V_{pclb26}) \times SBFF6P - (V_{pp14} \times SBFF6PQ) + (k_{as46} \times SWI4 \times SWI6PQ) - (k_{di46} \times SBFF6PQ) - (k_{asws} \times WHI5 \times SBFF6PQ) + (k_{diws} \times WSF6PQ) - ((ef4p \times V_{pclb}) \times SBFF6PQ) + (V_{pp14} \times SBFF46PQ) - (MSN5 \times SBFF6PQ)$

Differential Equations (cont.)

$\frac{d}{dt}SBFF46P = (k_{as46} \times SWI4P \times SWI6P) - (k_{di46} \times SBFF46P) + (ef4p \times V_{pclb}) \times SBFF6P - (V_{pp14} \times SBFF46P) - (V_{ppcln} \times SBFF46P) - ((ef6q \times V_{pclb26}) \times SBFF46P) + (V_{pp14} \times SBFF46PQ) + (ef4p \times V_{pclb}) \times SBFB6P + (ef6p \times V_{pcln}) \times SBFF4P$

$\frac{d}{dt}SBFF46PQ = (k_{as46} \times SWI4P \times SWI6PQ) - (k_{di46} \times SBFF46PQ) + (ef4p \times V_{pclb}) \times SBFF6PQ - (V_{pp14} \times SBFF46PQ) + (ef6q \times V_{pclb26}) \times SBFF46P - (V_{pp14} \times SBFF46PQ) + (ef4p \times V_{pclb}) \times SBFB6PQ - (MSN5 \times SBFF46PQ)$

$\frac{d}{dt}SBFB = (k_{asprom} \times SBFF \times PROM2) - (k_{diprom} \times SBFB) - (k_{asws} \times WHI5 \times SBFB) + (k_{diws} \times WSB) - ((ef6p \times V_{pcln}) \times SBFB) + (V_{ppcln} \times SBFB6P) - ((ef4p \times V_{pclb}) \times SBFB)$

$\frac{d}{dt}SBFB6P = (ef6p \times V_{pcln}) \times SBFB - (V_{ppcln} \times SBFB6P) + (k_{diwp} \times WSB56P) - ((ef6q \times V_{pclb26}) \times SBFB6P) + (V_{pp14} \times SBFB6PQ) - ((ef4p \times V_{pclb}) \times SBFB6P)$

$\frac{d}{dt}SBFB6PQ = (ef6q \times V_{pclb26}) \times SBFB6P - (V_{pp14} \times SBFB6PQ) - ((ef4p \times V_{pclb}) \times SBFB6PQ)$

$\frac{d}{dt}WSF = (k_{asws} \times WHI5 \times SBFF) - (k_{diws} \times WSF) - (k_{asprom} \times WSF \times PROM2) + (k_{diprom} \times WSB) - ((ef5p \times V_{pcln}W) \times WSF) + (V_{ppcln} \times WSF5P) - ((ef6p \times V_{pcln}) \times WSF) + (V_{ppcln} \times WSF6P) - ((ef4p \times V_{pclb}) \times WSF) + (V_{pp14} \times WSF4P)$

$\frac{d}{dt}WSF4P = (ef4p \times V_{pclb}) \times WSF - (V_{pp14} \times WSF4P) + (V_{ppcln} \times WSF46P) + (ef4p \times V_{pclb}) \times WSB - ((ef6p \times V_{pcln}) \times WSF4P) - ((ef5p \times V_{pcln}W) \times WSF4P)$

$\frac{d}{dt}WSF5P = (ef5p \times V_{pcln}W) \times WSF - (V_{ppcln} \times WSF5P) - (k_{diwp} \times WSF5P)$

$\frac{d}{dt}WSF6P = (ef6p \times V_{pcln}) \times WSF - (V_{ppcln} \times WSF6P) - ((ef5p \times V_{pclnw}) \times WSF6P) - ((ef6q \times V_{pclb26}) \times WSF6P) + (V_{pp14} \times WSF6PQ) + (k_{asws} \times WHI5 \times SBFF6P) - (k_{diws} \times WSF6P) - ((ef4p \times V_{pclb}) \times WSF6P) + (V_{pp14} \times WSF46P)$

$\frac{d}{dt}WSF6PQ = (ef6q \times V_{pclb26}) \times WSF6P - (V_{pp14} \times WSF6PQ) + (k_{asws} \times WHI5 \times SBFF6PQ) - (k_{diws} \times WSF6PQ) - ((ef4p \times V_{pclb}) \times WSF6PQ) + (V_{pp14} \times WSF46PQ) - (MSN5 \times WSF6PQ)$

$\frac{d}{dt}WSF45P = (ef4p \times V_{pclb}) \times WSB5P - (MSN5 \times WSF45P) + (ef5p \times V_{pclnw}) \times WSF4P$

$\frac{d}{dt}WSF46P = (ef4p \times V_{pclb}) \times WSF6P - (V_{pp14} \times WSF46P) - (V_{ppcln} \times WSF46P) - ((ef6q \times V_{pclb26}) \times WSF46P) + (V_{pp14} \times WSF46PQ) + (ef4p \times V_{pclb}) \times WSB6P + (ef6p \times V_{pcln}) \times WSF4P$

$\frac{d}{dt}WSF46PQ = (ef4p \times V_{pclb}) \times WSF6PQ - (V_{pp14} \times WSF46PQ) + (ef6q \times V_{pclb26}) \times WSF46P - (V_{pp14} \times WSF46PQ) + (ef4p \times V_{pclb}) \times WSB6PQ - (MSN5 \times WSF46PQ)$

$\frac{d}{dt}WSF56P = (ef5p \times V_{pclnw}) \times WSF6P - (k_{diwp} \times WSF56P)$

$\frac{d}{dt}WSB = (k_{asprom} \times WSF \times PROM2) - (k_{diprom} \times WSB) + (k_{asws} \times WHI5 \times SBFB) - (k_{diws} \times WSB) - ((ef5p \times V_{pclnw}) \times WSB) + (V_{ppcln} \times WSB5P) - ((ef6p \times V_{pcln}) \times WSB) + (V_{ppcln} \times WSB6P) - ((ef4p \times V_{pclb}) \times WSB)$

$\frac{d}{dt}WSB5P = (ef5p \times V_{pclnw}) \times WSB - (V_{ppcln} \times WSB5P) - ((ef6p \times V_{pcln}) \times WSB5P) + (V_{ppcln} \times WSB56P) - ((ef4p \times V_{pclb}) \times WSB5P)$

$\frac{d}{dt}WSB6P = (ef6p \times V_{pcln}) \times WSB - (V_{ppcln} \times WSB6P) - ((ef5p \times V_{pclnw}) \times WSB6P) + (V_{ppcln} \times WSB56P) - ((ef6q \times V_{pclb26}) \times WSB6P) + (V_{pp14} \times WSB6PQ) - ((ef4p \times V_{pclb}) \times WSB6P)$

Differential Equations (cont.)
$\frac{d}{dt}WSB6PQ = (ef6q \times V_{pclb26}) \times WSB6P - (V_{pp14} \times WSB6PQ) - ((ef4p \times V_{pclb}) \times WSB6PQ)$
$\frac{d}{dt}WSB56P = (ef6p \times V_{pcln}) \times WSB5P - (V_{ppcln} \times WSB56P) + (ef5p \times V_{pclnw}) \times WSB6P - (V_{ppcln} \times WSB56P) - (k_{diwp} \times WSB56P)$
$\frac{d}{dt}SWI6C = (MSN5 \times WSF45P) \times \frac{nucleus}{cytoplas} + (V_{pp14} \times SWI6QC) - (k_{imp} \times SWI6C)$
$\frac{d}{dt}SWI6QC = (V_{ppase} \times SWI6PQC) - (V_{pp14} \times SWI6QC)$
$\frac{d}{dt}SWI6PQC = (MSN5 \times SBFF6PQ) \times \frac{nucleus}{cytoplas} + (MSN5 \times SBFF46PQ) \times \frac{nucleus}{cytoplas} + (MSN5 \times WSF6PQ) \times \frac{nucleus}{cytoplas} + (MSN5 \times WSF46PQ) \times \frac{nucleus}{cytoplas} - (V_{ppase} \times SWI6PQC)$
$\frac{d}{dt}SWI6P = (ef6p \times V_{pcln}) \times SWI6 - (V_{ppcln} \times SWI6P) - ((ef6q \times V_{pclb26}) \times SWI6P) + (V_{pp14} \times SWI6PQ) - (k_{as46} \times SWI4 \times SWI6P) + (k_{di46} \times SBFF6P) - (k_{as46} \times SWI4P \times SWI6P) + (k_{di46} \times SBFF46P)$
$\frac{d}{dt}SWI6PQ = (ef6q \times V_{pclb26}) \times SWI6P - (V_{pp14} \times SWI6PQ) - (k_{as46} \times SWI4 \times SWI6PQ) + (k_{di46} \times SBFF6PQ) - (k_{as46} \times SWI4P \times SWI6PQ) + (k_{di46} \times SBFF46PQ)$
$\frac{d}{dt}MBFF = (k_{asmbf} \times MBP1 \times SWI6) - (k_{dimbf} \times MBFF) - (k_{asprom} \times MBFF \times PROM5) + (k_{diprom} \times MBFi)$
$\frac{d}{dt}MBFi = (k_{asprom} \times MBFF \times PROM5) - (k_{diprom} \times MBFi) - (V_{pclnm} \times MBFi) + (V_{ppcln} \times MBFa)$
$\frac{d}{dt}MBFa = (V_{pclnm} \times MBFi) - (V_{ppcln} \times MBFa) - ((k_{imbf01} \times CLB2) \times MBFa) + (k_{mbf10} \times MBFp) - ((k_{imbf02} \times NRM1) \times MBFa) + (k_{mbf20} \times MBFo) - (k_{aswm} \times MBFa \times WHI5) + (k_{diwm} \times WMB) + (ef5p \times V_{pclnw}) \times WMB$
$\frac{d}{dt}MBFp = (k_{imbf01} \times CLB2) \times MBFa - (k_{mbf10} \times MBFp) - ((k_{imbf02} \times NRM1) \times MBFp) + (k_{mbf20} \times MBFpo)$
$\frac{d}{dt}MBFo = (k_{imbf02} \times NRM1) \times MBFa - (k_{mbf20} \times MBFo) - ((k_{imbf01} \times CLB2) \times MBFo) + (k_{mbf10} \times MBFpo)$
$\frac{d}{dt}MBFpo = (k_{imbf01} \times CLB2) \times MBFo - (k_{mbf10} \times MBFpo) + (k_{imbf02} \times NRM1) \times MBFp - (k_{mbf20} \times MBFpo)$
$\frac{d}{dt}WMB = (k_{aswm} \times MBFa \times WHI5) - (k_{diwm} \times WMB) - ((ef5p \times V_{pclnw}) \times WMB)$
$\frac{d}{dt}WHI5PC = (MSN5 \times WHI5PN) \times \frac{nucleus}{cytoplas} + (MSN5 \times WSF45P) \times \frac{nucleus}{cytoplas} - (V_{pp14} \times WHI5PC)$
$\frac{d}{dt}WHI5C = (MSN5 \times WSF6PQ) \times \frac{nucleus}{cytoplas} + (MSN5 \times WSF46PQ) \times \frac{nucleus}{cytoplas} + (V_{pp14} \times WHI5PC) - (k_{imp} \times WHI5C)$
$\frac{d}{dt}WHI5PN = (ef5p \times V_{pclnw}) \times W4B + (ef5p \times V_{pclnw}) \times WHI5 - (V_{ppcln} \times WHI5PN) + (k_{diwp} \times WSF5P) + (k_{diwp} \times WSF56P) + (k_{diwp} \times WSB56P) - (MSN5 \times WHI5PN) + (ef5p \times V_{pclnw}) \times WMB$
$\frac{d}{dt}TCYCLE = 1.0$
$\frac{d}{dt}ORIFLAG = 0.0$

Differential Equations (cont.)
$\frac{d}{dt}Twhi5 = 0.0$
$\frac{d}{dt}Mwhi5 = 0.0$
$\frac{d}{dt}TBUD = 0.0$
$\frac{d}{dt}MBUD = 0.0$
$\frac{d}{dt}TORI = 0.0$
$\frac{d}{dt}UDNA = 0.0$
$\frac{d}{dt}REPDNA = 0.0$
$\frac{d}{dt}TSPN = 0.0$
$\frac{d}{dt}SPNALIGN = 0.0$
$\frac{d}{dt}SACOFF = 0.0$
$\frac{d}{dt}MASSBIRT = 0.0$
$\frac{d}{dt}MitCat = 0.0$

Implicit Species

Globals: Conditional Expressions
$global \quad -1 \quad CLB2 + CLB5 - KEZ2 - 0.0ORI = 0.0; ORIFLAG = 1.0$
$global \quad 1 \quad WHI5cycf - 0.5Twhi5 = TCYCLE; Mwhi5 = MASS$
$global \quad 1 \quad BUD - 1.0TBUD = TCYCLE; MBUD = MASS$
$global \quad 1 \quad ORI - 1.0 - 0.0TORI = TCYCLE \times ORIFLAG; UDNA = 1.0 \times ORIFLAG; MAD2 = mad2h \times ORIFLAG + mad2l \times (1.0 - ORIFLAG); BUB2 = bub2h \times ORIFLAG + bub2l \times (1.0 - ORIFLAG)$
$global \quad 1 \quad TCYCLE - TORI - DNATIMER - 0.0REPDNA = UDNA; SACOFF = SPNALIGN \times ORIFLAG; UDNA = 0.0; ORIFLAG = 0.0$
$global \quad 1 \quad TCYCLE - TORI - DNATIMER - 0.0MAD2 = mad2l \times SACOFF + mad2h \times (1.0 - SACOFF); BUB2 = bub2l \times SACOFF + bub2h \times (1.0 - SACOFF); LTE1 = lte1h \times SACOFF + lte1l \times (1.0 - SACOFF)$
$global \quad 1 \quad SPN - 1.0 - 0.0SPNALIGN = 1.0; TSPN = TCYCLE; SACOFF = REPDNA; MitCat = if(ESP1 > 0.1)then(1.0)else(0.0)$
$global \quad 1 \quad SPN - 1.0 - 0.0MAD2 = mad2l \times SACOFF + mad2h \times (1.0 - SACOFF); BUB2 = bub2l \times SACOFF + bub2h \times (1.0 - SACOFF); LTE1 = lte1h \times SACOFF + lte1l \times (1.0 - SACOFF)$

Globals: Conditional Expressions (cont.)
global -1 $CLB2-KEZ-0.0 MASS = F\times MASS\times REPDNA + MASS\times(1.0-REPDNA); LTE1 = lte1l\times REPDNA + LTE1\times(1.0-REPDNA); BUD = 0.0 + BUD\times(1.0-REPDNA); SPN = 0.0+SPN\times(1.0-REPDNA); TORI = 1000.0+TORI\times(1.0-REPDNA); TSPN = 0.0+TSPN\times(1.0-REPDNA); TBUD = 0.0+TBUD\times(1.0-REPDNA); Twhi5 = 0.0+Twhi5\times(1.0-REPDNA); MBUD = 0.0+MBUD\times(1.0-REPDNA); Mwhi5 = 0.0+Mwhi5\times(1.0-REPDNA); TCYCLE = 0.0; SACOFF = 0.0; SPNALIGN = 0.0; REPDNA = 0.0; MASSBIRT = MASS$

Initial Conditions

init $BUB2 = 0.2$

init $BUD = 0.0154110243593788$

init $C2 = 0.197341247757461$

init $C2P = 0.0128083052673014$

init $C5 = 0.0796549412241655$

init $C5P = 0.00476745860363818$

init $CDC14 = 0.685695353660066$

init $CDC15 = 0.652434722390971$

init $CDC15i = 0.347565277609012$

init $CDC20 = 0.706006222058708$

init $CDC20i = 0.787592468079176$

init $CDC6 = 0.133968163981639$

init $CDC6P = 0.035631491948781$

init $CDH1 = 0.996005362927922$

init $CDH1i = 0.00399463707207933$

init $CLB2 = 0.0694306531794237$

init $CLB5 = 0.0767894202634312$

init $CLN2 = 0.195608226760332$

init $ESP1 = 0.520542070433101$

init $F2 = 0.117541959300578$

init $F2P = 0.0280767101884002$

init $F5 = 1.04809529563752\times 10^{-4}$

init $F5P = 2.1402315582442\times 10^{-5}$

init $IE = 0.51615152340183$

init $IEP = 0.483848476598174$

init $LTE1 = 0.1$

init $MAD2 = 0.01$

init $MASS = 1.13467475183963$

init $MASSBIRT = 1.12860662640626$

$init \quad MBFa = 0.0$

$init \quad MBFF = 0.0$

$init \quad MBFi = 0.0$

$init \quad MBFo = 0.0$

$init \quad MBFp = 0.0$

$init \quad MBFpo = 0.0$

$init \quad MBP1 = 5.5$

$init \quad MBUD = 0.0$

$init \quad MitCat = 0.0$

$init \quad Mwhi5 = 0.0$

$init \quad NET1 = 0.00656818878723094$

$init \quad NET1P = 1.27912716487284$

$init \quad NRM1 = 0.0$

$init \quad ORI = 0.0291412146656156$

$init \quad ORIFLAG = 1.0$

$init \quad PDS1 = 0.0118584914876254$

$init \quad PE = 0.479457929566942$

$init \quad PROM2 = 2.0$

$init \quad PROM5 = 2.0$

$init \quad RENT = 0.643416336728071$

$init \quad RENTP = 0.870888309611862$

$init \quad REPDNA = 0.0$

$init \quad SACOFF = 0.0$

$init \quad SBFB6P = 0.0$

$init \quad SBFB6PQ = 0.0$

$init \quad SBFB = 0.0$

$init \quad SBFF46P = 0.0$

$init \quad SBFF46PQ = 0.0$

$init \quad SBFF4P = 0.0$

$init \quad SBFF6P = 0.0$

$init \quad SBFF6PQ = 0.0$

$init \quad SBFF = 0.0$

$init \quad SIC1 = 0.0368176074794449$

$init \quad SIC1P = 0.00518981723970555$

$init \quad SPN = 0.0325795571004625$

$init \quad SPNALIGN = 0.0$

$init \quad SWI4 = 5.5$

$init \quad SWI4B = 0.0$

$init \quad SWI4C = 0.0$

$init \quad SWI4F = 0.0$

$init \quad SWI4P = 0.0$

$\text{init} \quad SWI4PC = 0.0$

$init \quad SWI5 = 0.80343911939325$

$init \quad SWI5P = 0.0170776220724791$

$init \quad SWI6 = 30.0$

$init \quad SWI6C = 0.0$

$init \quad SWI6P = 0.0$

$init \quad SWI6PQ = 0.0$

$init \quad SWI6PQC = 0.0$

$init \quad SWI6QC = 0.0$

$init \quad TBUD = 0.0$

$init \quad TCYCLE = 0.72557268620405$

$init \quad TEM1GDP = 0.123253068346797$

$init \quad TEM1GTP = 0.876746931653158$

$init \quad TORI = 1000.0$

$init \quad TSPN = 0.0$

$init \quad Twhi5 = 0.0$

$init \quad UDNA = 0.0$

$init \quad W4B = 0.0$

$init \quad WHI5 = 10.0$

$init \quad WHI5C = 0.0$

$init \quad WHI5PC = 0.0$

$init \quad WHI5PN = 0.0$

$init \quad WMB = 0.0$

$init \quad WSB56P = 0.0$

$init \quad WSB5P = 0.0$

$init \quad WSB6P = 0.0$

$init \quad WSB6PQ = 0.0$

$init \quad WSB = 0.0$

$init \quad WSF45P = 0.0$

$init \quad WSF46P = 0.0$

$init \quad WSF46PQ = 0.0$

$init \quad WSF4P = 0.0$

$init \quad WSF56P = 0.0$

$init \quad WSF5P = 0.0$

$init \quad WSF6P = 0.0$

$init \quad WSF6PQ = 0.0$

$init \quad WSF = 0.0$

Parameters

$param \quad BCK2T = 0.075$

$param \quad bub2h = 1.0$

$param \quad bub2l = 0.2$

$param \quad cell = 1.0$

$param \quad CLN3T = 0.075$

$param \quad cytoplas = 0.8$

$param \quad DNATIMER = 20.0$

$param \quad ebudb5 = 0.5$

$param \quad ebudn2 = 0.28$

$param \quad ebudn3 = 0.25$

$param \quad ec1b2 = 0.4$

$param \quad ec1b5 = 0.23$

$param \quad ec1k2 = 0.0$

$param \quad ec1n2 = 0.09$

$param \quad ec1n3 = 0.0$

$param \quad ef4p = 1.0$

$param \quad ef5p = 1.0$

$param \quad ef6b2 = 1.5$

$param \quad ef6b5 = 0.45$

$param \quad ef6k2 = 0.0$

$param \quad ef6n2 = 0.23$

$param \quad ef6n3 = 0.0$

$param \quad ef6p = 1.0$

$param \quad ef6q = 1.0$

$param \quad eicdhb2 = 0.72$

$param \quad eicdhb5 = 4.2$

$param \quad eicdhn2 = 0.22$

$param \quad eicdhn3 = 0.0$

$param \quad eorib2 = 0.45$

$param \quad eorib5 = 0.85$

$param \quad epb5 = 0.07$

$param \quad epb5m = 0.16$

$param \quad epb5q = 1.0$

$param \quad epb5w = 0.075$

$param \quad epk2 = 0.56$

$param \quad epk2m = 0.61$

$param \quad epk2w = 0.3$

$param \quad epn2 = 0.083$

$param \quad epn2m = 0.04$

$param \quad epn2w = 0.08$

$param \quad epn3 = 1.0$

$param \quad epn3m = 1.0$

$param \quad epn3w = 1.0$

$param \quad J_{abck2} = 1.0$

$param \quad J_{acdh} = 0.03$

$param \quad J_{acln3} = 1.0$

$param \quad J_{aiep} = 0.1$

$param \quad J_{amcm} = 0.1$

$param \quad J_{atem} = 0.1$

$param \quad J_{d2c1} = 0.05$

$param \quad J_{d2f6} = 0.05$

$param \quad J_{ibck2} = 1.0$

$param \quad J_{icdh} = 0.032$

$param \quad J_{icln3} = 1.0$

$param \quad J_{iiep} = 0.1$

$param \quad J_{imcm} = 0.1$

$param \quad J_{item} = 0.1$

$param \quad J_{pn} = 0.4$

$param \quad J_{spn} = 0.15$

$param \quad k_{a15_p} = 0.002$

$param \quad k_{a15_{pp}} = 1.0$

$param \quad k_{a15_{ppp}} = 0.001$

$param \quad k_{a20_p} = 0.06$

$param \quad k_{a20_{pp}} = 0.15$

$param \quad k_{acdh_p} = 0.01$

$param \quad k_{acdh_{pp}} = 0.55$

$param \quad k_{aiep} = 0.1$

$param \quad k_{amcm} = 1.0$

$param \quad k_{as46} = 30.0$

$param \quad k_{asb2} = 50.0$

$param \quad k_{asb5} = 30.0$

$param \quad k_{asbf1} = 0.14$

$param \quad k_{asbf2} = 1.0$

$param \quad k_{asbf3} = 1.0$

$param \quad k_{asbf4} = 1.0$

$param \quad k_{asbf5} = 0.11$

$param \quad k_{asesp} = 50.0$

$param \quad k_{asf2} = 15.0$

$param \quad k_{asf5} = 0.015$

$param \quad k_{asmbf} = 30.0$

$param \quad k_{asprom} = 50.0$

$param \quad k_{asrent} = 300.0$

$param \quad k_{asrentp} = 1.0$

$param \quad k_{asw4} = 0.0$

$param \quad k_{aswi} = 2.0$

$param \quad k_{aswm} = 1.5$

$param \quad k_{asws} = 30.0$

$param \quad k_{d14} = 0.1$

$param \quad k_{d1c1} = 0.01$

$param \quad k_{d1f6} = 0.01$

$param \quad k_{d20} = 0.255$

$param \quad k_{d20_p} = 0.05$

$param \quad k_{d2c1} = 1.0$

$param \quad k_{d2f6} = 1.0$

$param \quad k_{d3c1} = 1.0$

$param \quad k_{d3f6} = 1.0$

$param \quad k_{db2_p} = 0.003$

$param \quad k_{db2_{pp}} = 0.4$

$param \quad k_{db2_{ppp}} = 0.285$

$param \quad k_{db5_p} = 0.015$

$param \quad k_{db5_{pp}} = 0.17$

$param \quad k_{dbs4} = 5.0$

$param \quad k_{dbud} = 0.06$

$param \quad k_{dc1} = 0.001$

$param \quad k_{dcdh} = 0.01$

$param \quad k_{df6} = 0.001$

$param \quad k_{di46} = 0.5$

$param \quad k_{dib2} = 0.05$

$param \quad k_{dib5} = 0.06$

$param \quad k_{diesp} = 0.5$

$param \quad k_{dif2} = 0.5$

$param \quad k_{dif5} = 0.03$

$param \quad k_{dimbf} = 1.0$

$param \quad k_{diprom} = 1.0$

$param \quad k_{dirent} = 1.0$

$param \quad k_{direntp} = 1.6$

$param \quad k_{diw4} = 1.0$

$param \quad k_{diwm} = 1.0$

$param \quad k_{diwp} = 15.0$

$param \quad k_{diws} = 1.0$

$param \quad k_{dn2} = 0.14$

$param \quad k_{dnet} = 0.03$

$param \quad k_{dnrm1} = 0.08$

$param \quad k_{dori} = 0.06$

$param \quad k_{dpd_{0001}} = 0.03$

$param \quad k_{dpds_p} = 0.01$

$param \quad k_{dpds_{pp}} = 0.25$

$param \quad k_{dspn} = 0.06$

$param \quad k_{dswi} = 0.08$

$param \quad k_{EZ2} = 0.2$

$param \quad k_{EZ} = 0.3$

$param \quad k_{galbck2} = 3.0$

$param \quad k_{galcdc6} = 0.4$

$param \quad k_{galclb2} = 0.38$

$param \quad k_{galclb5} = 0.016$

$param \quad k_{galcln2} = 0.165$

$param \quad k_{galcln3} = 15.0$

$param \quad k_{galsic1} = 0.132$

$param \quad k_{gk_{cln3}} = 1.0$

$param \quad k_{i15} = 0.5$

$param \quad k_{i} = 40.0$

$param \quad k_{icdh_p} = 0.005$

$param \quad k_{icdh_pp} = 0.08$

$param \quad k_{iiep} = 0.15$

$param \quad k_{imbf01} = 0.6$

$param \quad k_{imbf02} = 1.2$

$param \quad k_{imcm} = 0.49$

$param \quad k_{imp} = 4.0$

$param \quad k_{iswi} = 0.1$

$param \quad k_{mbf10} = 0.12$

$param \quad k_{mbf20} = 0.12$

$param \quad k_{mcbck_2} = 5.0$

$param \quad k_{mccdc6} = 5.0$

$param \quad k_{mcclb5} = 4.0$

$param \quad k_{mccln2} = 4.0$

$param \quad k_{mccln3} = 15.0$

$param \quad k_{mcsic1} = 4.0$

$param \quad k_{mcwhi5} = 10.0$

$param \quad k_{p_p} = 0.01$

$param \quad k_{p_pp} = 1.0$

$param \quad k_{pnet_p} = 0.01$

$param \quad k_{pnet_pp} = 2.4$

$param \quad k_{pp14} = 1.0$

$param \quad k_{pp} = 0.1$

$param \quad k_{ppc1} = 4.0$

$param \quad k_{ppcln} = 1.0$

$param \quad k_{ppcln_p} = 0.5$

$param \quad k_{ppf6} = 4.0$

$param \quad k_{ppn_{0001}} = 2.8$

$param \quad k_{ppnet_p} = 0.05$

$param \quad k_{s14} = 0.22$

$param \quad k_{s20_p} = 0.006$

$param \quad k_{s20_{pp}} = 0.6$

$param \quad k_{sb2_p} = 0.006$

$param \quad k_{sb2_{pp}} = 0.12$

$param \quad k_{sb5_p} = 2.0 \times 10^{-4}$

$param \quad k_{sb5_{pp}} = 0.004$

$param \quad k_{sb5_{ppp}} = 0.04$

$param \quad k_{sbs4} = 5.0$

$param \quad k_{sbud} = 0.2$

$param \quad k_{sc1_p} = 0.0132$

$param \quad k_{sc1_{pp}} = 0.132$

$param \quad k_{scdh} = 0.01$

$param \quad k_{sf6_p} = 0.02$

$param \quad k_{sf6_{pp}} = 0.2$

$param \quad k_{sf6_{ppp}} = 0.004$

$param \quad k_{sn2_p} = 0.0$

$param \quad k_{sn2_{pp}} = 0.38$

$param \quad k_{sn2_{ppp}} = 0.3$

$param \quad k_{snet} = 0.084$

$param \quad k_{snrm1_p} = 0.08$

$param \quad k_{sori} = 1.5$

$param \quad k_{spds_p} = 0.07$

$param \quad k_{ssa0} = 0.6$

$param \quad k_{ssab2} = 0.5$

$param \quad k_{ssaw5} = 7.0$

$param \quad k_{sspn} = 0.09$

$param \quad k_{sswi_p} = 0.005$

$param \quad k_{sswi_{pp}} = 0.08$

$param \quad k_{ydj1} = 1.0$

$param \quad lte1h = 1.0$

$param \quad lte1l = 0.1$

$param \quad mad2h = 8.0$

$param \quad mad2l = 0.01$

$param \quad maxmass = 28.0$

$param \quad mdt = 90.0$

$param \quad msn5 = 25.0$

$param \quad n = 5.0$

$param \quad nucleus = 0.2$

$param \quad pp2at = 1.0$

$param \quad ppase = 1.0$

$param \quad vnmaxw = 3.0$

$param \quad vpmaxm = 5.8$

$param \quad vpnmax = 3.0$

$param \quad whi5op = 10.0$

ODE modeler/simulator parameters

$dt = 1.0, total = 500.0, maxstor = 50000, bound = 10000, toler = 1.0 \times 10^{-4}, njmp = 1, t_0 = 0.0, meth = stiff$
