# Supplementary material for: Modeling the START transition in the budding yeast cell cycle
Source: PLoS Comput Biol. 2024 Aug 2;20(8):e1012048. doi: 10.1371/journal.pcbi.1012048 (PMC11324117; doi:10.1371/journal.pcbi.1012048)
Supplement: Attachment — Submitted filename: START-Response_to_reviewers.pdf [file pcbi.1012048.s018.pdf]

Response to Reviewers

First and foremost, we sincerely appreciate the positive feedback and encouraging comments of the reviewers. The points raised have been critical in making the manuscript and accompanying data complete to ensure all key aspects of the START transition in the budding yeast cell cycle have been adequately addressed in the model and manuscript. As the reviewers have acknowledged, this is a very detailed and complex mathematical model that we have constructed to recapitulate this critical transition. We are excited that the model emulates and matches experimental data both qualitatively and quantitatively the majority of the time, including 150+ mutant phenotypes. We have acknowledged and included (both in the past and now in response to reviewers) any limitations that the model may have, given the multidimensionality of the data fitting problem we face. Also, in some cases, if the variables are outside START or affecting START, albeit through factors in the model, we've modeled and parameterized the indirect effects, if not explicitly (as addressed below). We hope the manuscript in its current revised form (changes highlighted) will be satisfactory to the reviewers and that the cell cycle and modeling fields benefit from this contribution. There are also additional changes in the manuscript (including changes to Fig. 5 and license, adding Supplementary legend to the main text) made upon request from the editorial staff.

Comments to the Authors:
Please note here if the review is uploaded as an attachment.

Reviewer #1: The article by Ravi et al describes a new version of the cell cycle model that was initially developed many years ago for budding yeast cells and pioneered the powerful application of systems biology to a complex biological process. This model has been subject to periodic revisions to accommodate the new knowledge attained in this active field of research and, in this occasion, the authors focus their attention to the molecular interactions that control START in late G1. As in prior versions, the model is very well developed and tested, and the needed assumptions are reasonable in general terms. There are, nevertheless, important experimental data that should be adopted by the model to attain a more realistic description of the triggering factors of START. In addition, some issues should be clarified or solved in the text.

We appreciate the reviewer's positive comments and thank the reviewer for their attention to detail. The detailed feedback, we believe, has made the manuscript even more comprehensive. We hope the changes made in the main text (and below) address the concerns raised in full.

1. "In early G1, only Cln3 is available, and its level increases with cell size (ref. 2)". This is still a matter of debate and refs for experimental evidence in favor and against should be provided. Please also check ref 2.

This is a fair point to establish clearly the state of the field. As suggested, we have now modified the language to highlight multiple proposed mechanisms currently debated for Cln3 concentration and regulation, and provided references to competing viewpoints. We have corrected the citation of Ref.2 in this context.

2. Dilution of Whi5 in G1 has been proposed as a relevant sizer at START (10.1038/nature14908). This mechanism should be underlined in the text and, preferably, added to the model. Also, in table S2 the authors state that "Whi5 is known to be transcriptionally regulated showing a 3-fold variation, peaking

in late G1 phase (ref. 4)". By contrast, WHI5 is more expressed in G2/M phases (10.1038/nature14908).
We have added discussion and references for the role of Whi5 dilution as cell size increases and corrected the timing of peak Whi5 expression in Table S2. In model scenarios with increasing Whi5 concentrations, we see a minor increase in cell mass, and with decreasing Whi5 concentrations, daughter cells are predicted to show iteratively less mass over generations, supporting that its putative role as a sizer is already represented. Work by Barber, Amir, and Murray (10.1073/pnas.2001255117), as well as by Sommer *et al*. (10.7554/elife.64364), also experimentally demonstrated only modest increases in cell size with Whi5 overexpression.

3. "Ydj1 is believed to act as the cell size sensor". However, Ssa1 and Ydj1 chaperones have been involved in cell size control as growth rate sensors (ref. 79), not as reporters of size per se. In addition, in table S2 the authors state that "Ydj1, which is proposed to be the sensor of cell size (ref. 18)". Please check the reference and clarify.
We have modified the text to better convey the more nuanced roles of Ydj1 and Ssa1. We model Ydj1 as a function of mass, and the mechanisms underlying this are related to growth rate. Indeed, our model uses cell mass as a proxy for cell size, but growth rate vs. cell size are clearly distinct. We have adjusted language for clarity in this case as well. Supplemental reference 18 (Vergés *et al*., now reference 19 post-revisions) is the same as the main manuscript's reference 79 (previously 79, now 84 post-revisions), and we have adjusted the language about "cell size" to more accurately reflect growth rate instead. Additionally, we have now made this clear distinction in both the Results section and figure legends when they first appear.

4. Degradation of Cln3 by SCF-Cdc4/Mad3 has been shown to cause a progressive accumulation of Cln3 in the nucleus during G1 (10.1126/sciadv.abm4086). As this mechanism connects START to the preceding cell cycle, it should be included in the model as a sizer mechanism. In addition, the authors could test whether activation of the spindle checkpoint predicts a larger cell size in the subsequent START event as observed.
We have incorporated the Pérez et al. reference and the influence of SCF/Cdc4/Mad3 as additional mechanisms of regulation. Whether the effects of other factors are ubiquitination or sequestration of Cln3 anywhere outside of the nucleus, the effect on the model is ultimately determined by Cln3 concentrations, which can be adjusted as desired through the deactivation parameter. While additional mechanisms both in and outside of START do have roles, we have opted to minimize the number of modeled features when possible to avoid overfitting. Nevertheless, we have increased mad2h and bub2h parameters to simulate spindle checkpoint activation and do see a marginal increase in cell mass predicted (below).

5. Although not strictly required in the model, RNAPol2 phosphorylation by Cdc28/Cln3 should be considered in the text.
We agree and have added the proposed role of Cln3-Cdk1 in acting upon RNA polymerase 2 activity through Rpb1 for SBF activity. We mention that the typically proposed role of Whi5 phosphorylation may work as a separate input with Cln3 through Rpb1 to act on SBF.

Reviewer #2: In the manuscript entitled 'Modeling the START transition in the budding yeast cell cycle' by Ravi et al, the authors proposed a detailed dynamical model of START transition in the Saccharomyces cerevisiae cell cycle. Their model of START transition is integrated into the well-established previous model of budding yeast cell cycle (Chen et al, Mol. Biol. Cell 15, 3841(2004)).

The model takes into account key details of activation, inactivation and localization of several regulatory factors Swi4, Swi6, SBF, Whi5, MBF and Bck2 to explain key mutant phenotypes associated with the START transition in the budding yeast cell cycle. The model indeed captures phenotypic behavior of many mutants relevant to the START transition.

The manuscript has great merit for publication in the PloS Computational Biology. I have a few queries and suggestions for the authors to consider in their revision.

We appreciate the reviewer's appreciative notes and positive comments. We have provided additional clarification, and supporting data files to help with the manuscript revisions. We hope these address all the concerns raised.

1. For a mathematical model paper, it is extremely important to have the model equations listed in a table either in the main or supplementary text. These equations should be listed in the readable format in a table instead of .ode or .pet format as supplementary file. Supplementary files (.ode or .pet) are required for reproducibility.

In addition to providing the model files for reproducibility, we have provided the equations in a tabular format (PDF) as requested.

2. Single cell quantitative experiments on budding yeast cell cycle have shown that the average cycle time of daughter and mother cells, respectively, are 112 and 87 min in glucose medium for the WT cells (Di Talia et al, Nature 448, 947 (2007)). In addition, the average duration of the unbudded phase for them were found to be 37 and 16 min, respectively. Although the model nearly recaptures the cell cycle duration (107 min) of the daughter cell however it reports a significantly longer duration of G1 phase (62 min) and consequently the SG2M phase (or budded phase) becomes quite short. Therefore the authors need to justify the gap between the model and the available experimental data. Furthermore the details of mother-daughter distinction at the cell division need to be mentioned.

We acknowledge that, while total cell cycle times are very near experimentally validated measurements, the model prediction for G1 duration is different from the experimental values. The G1 phase for daughter cells is predicted to be 53 minutes, which is ~15min longer than expected based on experimental observations. However, the ability of the simulation to correctly predict (qualitatively and quantitatively) the behaviors of numerous experimentally determined mutant phenotypes, along with tracking relevant molecular variables, shows that the model robustly emulates the biological sequence of START events in the cell cycle and resulting outcomes (phenotypes such as viability, relative size) and adds significant value in most contexts, but for precise timings. We have now mentioned this as a limitation in the main text, too. Additionally, we have ensured the text does not overstate the ability of our model to recapitulate precise timings for G1 or budded phases.

We have also added clarifying details for mother-daughter distinction (e.g., that we follow daughter cells by default, and how tracking parent cells could be achieved by the model) at cell division as suggested.

3. Previous experimental (Schmoller et al, Nature 526, 268 (2015)) and modeling (Heldt at al, PLoS Comput Biol 14(10): e1006548 (2018)) studies have uncovered a new perspective of size control in budding yeast cell cycle. According to these studies dilution of cell cycle inhibitor (Whi5) and titration of cell cycle activator (Cln3) become key driving factor for the size control in budding yeast. It will be

worth discussing the feasibility and connection of dilution-titration mechanism in this model.

We agree that the dilution-titration model is worth detailing, and we have now added language to the main text that outlines these findings and compares our results with the expectations of the dilution-titration framework. In our model, Whi5/Cln3 (or other variables) concentrations can be tuned with the corresponding parameters, including the multicopy parameters that allow mimicking deletion, overexpression, or titration studies.

4. The Figure S5 gives a false impression of mismatch between the MDT and the cell cycle time, as it plots the cell cycle time of the daughter cell with the MDT of the population consisting of both mother and daughter cells.

The original data from Lord and Wheals (1980) plots the duration of daughter and parent, budding, and parent-to-bud period against doubling time (Lord and Wheals, Fig. 1). Therefore, the doubling time on the x-axis includes both parent and daughter cell populations. In Figure S5, we only plot the daughter timings: total cell cycle, G1, and budded times. While cycle times move concurrently with the mass doubling times in experiments, we note in the figure legend that the simulated budded cycle times differ from experimental counterparts, touching on the model differences we discuss in comment #2. However, if this figure with daughter data distracts from the overall story and its import rather than providing a useful comparison, we are happy to remove it at further reviewer request.

5. For better readability, the authors may think about alternative ways of reporting the results of Figure 8 as the plots spread across multiple pages.

We agree, and to address this, we have created 3x5 plots to avoid the spread across multiple pages. Other multi-page main figures have been condensed likewise.

Have the authors made all data and (if applicable) computational code underlying the findings in their manuscript fully available?

The PLOS Data policy requires authors to make all data and code underlying the findings described in their manuscript fully available without restriction, with rare exception (please refer to the Data Availability Statement in the manuscript PDF file). The data and code should be provided as part of the manuscript or its supporting information, or deposited to a public repository. For example, in addition to summary statistics, the data points behind means, medians and variance measures should be available. If there are restrictions on publicly sharing data or code —e.g. participant privacy or use of data from a third party—those must be specified.

Reviewer #1: Yes

Reviewer #2: Yes
